# Preferences for characteristics of contraceptive methods: a large, multi-country discrete choice experiment

**DOI:** 10.1136/bmjgh-2025-023196

**Published:** 2026-08-24

**Authors:** Hae-Young Kim, Fred Church, LinChiat Chang, Della Berhanu, John Kinuthia, Anna Bershteyn

**Affiliations:** 1 Department of Population Health, New York University Grossman School of Medicine, New York, New York, USA; 2 Format Analytics, Rockaway, New Jersey, USA; 3 Independent consultant, Cape Town, South Africa; 4 Ethiopian Public Health Institute, Addis Ababa, Ethiopia; 5 London School of Hygiene & Tropical Medicine, London, UK; 6 Department of Research & Programs, Kenyatta National Hospital, Nairobi, Kenya

**Keywords:** Africa, Global Health, Kenya, Cross-sectional survey, Pregnancy

## Abstract

**Background:**

Nearly half of global pregnancies are unintended. Contraceptive use is preference-sensitive, including concerns about convenience, privacy and side effects (eg, irregular bleeding, weight gain). We conducted a multi-country study examining women’s preferences for attributes of current and potential future modern hormonal contraceptives using a discrete choice experiment (DCE).

**Methods:**

Between April and October 2022, we recruited nationally representative samples of women of reproductive age from seven countries (Burkina Faso, Nigeria, Ethiopia, Kenya, Zambia, Pakistan and the USA) using multi-stage random cluster sampling from national census enumeration areas. The DCE comprised six attributes of hormonal contraceptives: format (ie, mode of administration: injection, implant, etc), dosing interval, provider-administration versus self-administration, effect on menstruation, effect on weight and time from discontinuation until return to fertility. Mixed logit models were fitted separately by country to estimate the relative preference weights (β) for each attribute level.

**Results:**

Across 20 068 participants, format was the strongest driver of preferences. Injectables were most preferred in Kenya (β=0.44, 95% CI 0.25 to 0.63), Pakistan (β=0.55, 95% CI 0.29 to 0.81), Ethiopia (β=0.64, 95% CI 0.38 to 0.90) and Zambia (β=0.39, 95% CI 0.20 to 0.58). Oral pills were most preferred in the USA (β=0.78, 95% CI 0.58 to 0.98), Burkina Faso (β=0.43, 95% CI 0.27 to 0.59) and Nigeria (β=0.55, 95% CI 0.40 to 0.70). Intrauterine devices and vaginal rings were less preferred in all countries. Menstrual bleeding changes and dosing interval were secondary drivers, with longer-acting methods modestly preferred and ongoing irregular bleeding modestly non-preferred. Avoiding weight gain was significantly preferred in several countries, most strongly in Pakistan (β=0.56, 95% CI 0.44 to 0.67) and the USA (β=0.42, 95% CI 0.31 to 0.53).

**Conclusions:**

In this large, multi-country study, reproductive-age participants expressed strong, heterogeneous preferences for contraceptive format, and in some settings, avoiding weight gain side effects. These findings can support preference-aligned contraceptive product development.

**WHAT IS ALREADY KNOWN ON THIS TOPIC**

- We searched PubMed and Google Scholar for articles published in English up to 15 June 2025, using the search terms ‘contraceptive preferences,’ ‘discrete choice experiment’ and ‘preference measurement.’
- Prior studies using discrete choice experiments (DCEs) to assess contraceptive preferences relied on convenience sampling rather than a nationally representative, census-driven sample. Most were conducted in a single country or sub-national region and included a relatively small number of study participants (under 500).
- Most focused on a single contraceptive product format (eg, implants or injections) or examined a narrow set of product attributes, limiting the ability to compare the importance of contraceptive formats and attributes across different settings.

**WHAT THIS STUDY ADDS**

- To our knowledge, this study provides the most geographically extensive, nationally representative information to date on women’s preferences for contraceptive product attributes, including over 20 000 women from seven countries (Burkina Faso, Nigeria, Ethiopia, Kenya, Zambia, Pakistan and the USA).
- Using a standardised, pre-tested DCE, we quantified how women make trade-offs across product format (ie, mode of administration), dosing interval, time from discontinuation until return to fertility and side effects (menstrual bleeding and weight gain).
- We found that the contraceptive format was the most influential driver of preference, followed by effects on menstruation and weight gain. While preferences for method format varied across countries, substantial heterogeneity within countries highlights the importance of offering a method mix that aligns with the spectrum of local needs and preferences.

**HOW THIS STUDY MIGHT AFFECT RESEARCH, PRACTICE OR POLICY**

- Across all countries, we found that contraceptive format and side effects are the most critical factors shaping women’s preferences.
- Programmes seeking to expand modern contraception should prioritise not only contraceptive availability and affordability, but also alignment of available methods with user preferences, particularly with regard to product format and tolerability of side effects.
- Further research is needed to understand time-varying preferences, including the effect of increasing women’s familiarity with newer contraceptive formats.

## Introduction

 Nearly half of pregnancies worldwide are unintended, totalling 121 million each year.^1 2^ Over 80% of these pregnancies occur among women not using contraception, with another 10% using a traditional method rather than a modern method.^3^ Expanding access to and use of modern contraceptive methods could reduce unintended pregnancies by 68% and maternal mortality by up to 23%.^4^ However, despite these potential benefits, many women do not use modern contraceptive methods, more often due to socio-cultural beliefs or norms^5 6^ and undesirable product characteristics than because of structural barriers such as availability and costs.^7 8^ To address these barriers, contraceptive product developers are advancing research and development of new contraceptives spanning a wide range of product formats,^9^ including next-generation vaginal rings,^10^ biodegradable implants,^11^ microneedle array dermal patches^12^ and longer-acting injections.^13 14^ The benefits of these innovations will depend in large part on whether their design facilitates uptake and effective use, especially for those who would otherwise be at risk of unintended pregnancy.

Contraceptive method mix varies widely across settings and population subgroups,^15^ in many cases appearing to be driven more by preferences than by availability of contraceptive options.^16^ For example, oral contraceptive pills are the most-used reversible method in the USA,^17^ while injectables are the most-used method in the sub-Saharan Africa region^18^—despite the wide availability of oral contraceptives in low-income and middle-income countries (LMICs) and injections in high-income countries.^18^ However, usage patterns are not always a reliable measure of preference, as they can be conflated with subtleties of access. For example, national-level product registration and distribution does not guarantee local availability—especially in remote areas. Stock-outs can make methods intermittently unavailable. Healthcare providers may lack time to present all available options to clients. Therefore, to measure end-users’ preferences independently of access considerations, more controlled preference studies can usefully complement observed method mix.

Discrete choice experiments (DCE) are a well-established health economic and service research methodology for quantifying preferences. They involve participants making choices among options with embedded trade-offs. DCEs are based on random utility theory, which assumes that individuals are rational decision-makers and select the choice that maximises their utility.^19^ Several studies have used DCEs to study preferences for contraceptive methods, considering factors such as the extent to which contraceptive methods can be used privately (ie, without inadvertent disclosure to others), how soon fertility returns after discontinuation, and side effects such as bleeding changes and weight gain.^4^ Among adolescent girls and young women in Kenya, bleeding pattern was the single most important driver of contraceptive preference, with unchanged bleeding most preferred, irregular bleeding less preferred, and heavier bleeding least preferred.^20^ In Nigeria and India, preferences for a potential microarray patch were most strongly influenced by effect on menstrual bleeding and dosing interval.^21^ In an urban informal settlement in Ghana, both preferences and use of contraceptives were strongly associated with method reversibility and minimising side effects such as impact on blood pressure.^22^

While informative, prior studies were limited by small sample sizes (typically fewer than 500 study participants) with narrow geographic scopes, and were often convenience samples and/or studies of a specific population segment or community. Moreover, many studies either focused on a specific contraceptive format^21^ or did not include the product format as an attribute,^20 22^ limiting the ability to assess trade-offs for attributes within and across contraceptive formats.

Here, we present what is to our knowledge the largest study to date quantifying preferences for a wide range of contraceptive attributes, including format, across seven countries. Study countries spanned three continents and the full spectrum of country income groups, with particularly strong representation from sub-Saharan Africa—the global region with the highest rates of unintended pregnancy^23^ and maternal mortality.^24^ Participants were nationally representative, with sampling guided by each country’s national census. By using a common survey in all countries, we aimed to produce robust, representative data that could inform both local family planning implementation and global-scale development of next-generation contraceptive methods.

## Methods

### Study design and settings

We conducted a multi-country DCE with a standardised experimental design implemented across seven countries: Burkina Faso, Nigeria, Ethiopia, Kenya, Zambia, Pakistan and the USA. These countries span diverse geographic regions and income groups. The five SSA countries were selected due to high burdens of unintended pregnancy^23^ and maternal mortality,^24^ diverse contraceptive method mixes and ongoing investments in family planning programmes.^25^ Pakistan was included to represent a South Asian setting with substantial unmet need for contraception,^26^ while the USA represented a high-income country with access to a broad range of contraceptives. Together, these settings capture variation in demographic, socio-economic and programmatic contexts to inform product development for global deployment.

### Eligibility

Eligible participants (1) reported being able to become pregnant, (2) reported having sex with a male partner in the past 12 months and (3) fell within age eligibility for the corresponding country. In Pakistan, based on cultural feedback obtained during formative and pilot research, the second criterion was expanded to reporting being married. Age eligibility was adjusted from the initial target of 15–49 to respect local cultures and norms. In the final survey, eligible ages were 15–49 in Kenya, Nigeria and Burkina Faso; 16–49 in Pakistan; and 18–49 in Ethiopia, Zambia and the USA.

### Selection of contraceptive product features for the choice experiment

Between January and March 2022, we used a triangulated process to identify the most important features of emerging modern contraceptive methods. First, we consulted contraceptive product developers to map key attributes of current and pipeline hormonal products. Second, we conducted a literature review on prior DCE studies and acceptability and preferences for contraceptives to identify attributes that have consistently emerged as important to end-users. Third, during formative work and pretesting in each country, respondents and local teams provided feedback on the relevance, wording and plausibility, which informed refinement of the final attribute set and their presentation. The final set of attributes (table 1) included format, dosing interval, whether products could be self-administered and/or administered by a healthcare provider, effect on menstruation, effect on weight and time from discontinuation until return to fertility.

**Table 1:** Attributes and levels in the discrete choice experiment assessing hormonal contraceptive method preferences

Format	Pill	Injection	Implant	Micro-array patch	IUD	Vaginal ring
Dosing interval	24 hours before/after sex	3 months	6 months	3 months	6 months	1 month
	1 month	4 months	1 year	4 months	2 years	3 months
	3 months	6 months	2 years	6 months	5 years	1 year
Who administers	Self	HCW* or self	HCW	HCW or self	HCW	HCW or self
		Self		Self		Self
		HCW		HCW		HCW
Effect on menstruation	The same as your current bleeding					
	Irregular bleeding for 3 months, then regular bleeding for 1 week every month					
	Irregular bleeding for 3–6 months, then regular bleeding for 1 week every month					
	Irregular bleeding for 3–6 months, then no bleeding					
	Irregular bleeding for as long as the product is used					
Effect on weight	No change					
	You may gain up to 2.5 kg in 1 year					
	You may gain up to 4.5 kg in 1 year					
Return to fertility†	1 month	1 month	1 month	1 month	1 month	1 month
	2 months	3 months	2 months	3 months	2 months	2 months
	3 months	6 months	3 months	6 months	3 months	3 months
	6 months	12 months	6 months	12 months	6 months	6 months
			12 months		12 months	
			18 months		18 months	

* HCW (healthcare workers) refers to a trained healthcare provider. Levels shown for each attribute vary by method (ie, format) for duration, administration and return to fertility.

† Levels of return to fertility were chosen based on the dosing interval. For each dosing interval, three levels of return to fertility were available to choose from. See S6 in detail.

HCW, healthcare worker; IUD, intrauterine device.

Levels of each attribute were determined based on expert consultation and landscaping of contraceptive products and research. Formats included were pills, injectables, implants, intrauterine devices (IUDs), vaginal rings and micro-array patches. Attribute levels were selected to represent a plausible range for each product, including concepts under development. Provider-administration and self-administration options differed by format. IUDs and implants were always provider-administered, pills were always self-administered, and all other formats were presented with three levels: provider-administered, self-administered, or able to be either provider-administered or self-administered. Dosing interval and return-to-fertility rates also differed by format (online supplemental table S1). All formats had short, medium and long dosing intervals, but the intervals differed by format (table 1, second row). Similarly, return-to-fertility rates had three levels with different values depending on format (table 1, bottom row).

These format-specific levels were chosen to avoid implausible or illogical combinations of attributes (for example, an IUD that requires daily administration) and therefore introduced structured dependence among format, dosing interval and return-to-fertility. Preference weight estimates for dosing interval and return-to-fertility therefore reflect utilities over the feasible, format-appropriate ranges presented in the experiment, rather than fully independent effects applicable across formats.

### DCE design

For oral pill, IUD and implant formats, there were 135 unique profiles (3 frequencies of administration × 3 return-to-fertility times × 3 effects on weight × 5 effects on menstrual bleeding). For injection, micro-array patch and vaginal ring formats, there were three times more possibilities because each method could be self-administered, provider-administered or be administrable either way. Across formats, the DCE had 1620 possible combinations, far too many to present to a single respondent.

Therefore, we used the *AlgDesign* R package to generate a D-optimal design from the full factorial space. We first specified the candidate set of all feasible profiles, respecting the format-specific constraints on dosing interval and return-to-fertility. We then used the *optFederov* function to identify a design that maximised expected parameter precision (D-efficiency) and supported good prediction performance (I-efficiency). The selected design consisted of 23 distinct choice cards that provided broad coverage of attribute levels and low collinearity.

We then augmented this design with seven additional choice cards that represented specific product profiles of contraceptive concepts under development as of March 2022. These ‘concept cards’ were included to directly inform policy and product development for prioritised formats and attribute combinations. Although augmenting an optimised design with additional tasks can modestly reduce statistical efficiency, including these cards allowed explicit estimates for the most policy-relevant product profiles. The final DCE design contained 30 choice cards, such as the example shown in online supplemental figure S1. We assessed the final design for approximate balance in level frequencies and minimal overlap across attributes.

DCEs typically include a set of 8 to 16 choice tasks for each respondent, which gives a reasonable balance between capturing preferences and avoiding respondent burden and fatigue.^19^ To reduce cognitive burden, we created three balanced blocks of 10 choice cards each, and each respondent was randomly assigned to one block and completed 10 choice tasks. Each choice card presented two product profiles (Product X and Y) and a third opt-out option (‘neither’).

DCEs were translated into local languages using a forward-backward translation process. Local teams reviewed translated versions to ensure conceptual equivalence and independently reverse-translated. We developed visual aids to depict each product format, mode of administration, and approximate site of administration, and these were iteratively refined in collaboration with local partners. Cognitive pretests and pilot interviews were conducted in each country to evaluate comprehension of the DCE instructions, attribute descriptions and choice tasks. Feedback from participants and interviewers was used to refine translations and ensure understanding of key survey questions and the DCE choice cards. A laminated reference sheet that displayed all product formats and their visual representations was available to respondents throughout survey completion.

### Sample size and sampling strategy

We aimed to recruit nationally representative samples and obtain robust national estimates in the presence of heterogeneity. We also aimed to support future sub-national analyses with up to 15 geospatial or sub-population segments in each country. Power analysis resulted in a recruitment target of 3000 participants per country (online supplemental appendix S1).

In all countries except for the USA, we used national census data to implement multi-stage random cluster sampling with probability proportional to size, stratified by urban versus rural area, to produce a set of census enumeration areas designated as the study’s primary sampling units. In the USA, we employed a hybrid sampling design with both probability-based and non-probability web panels, with recruitment calibrated to the US census to adjust for self-selection bias in the non-probability sample. All samples were country-wide except for Ethiopia, where only the south of the country was included in the study, as it was not safe for research teams to visit the north due to war. Additional details of participant sampling are provided in online supplemental appendix S2.

### Data collection

Interviewers completed 2–3 days of training covering household sampling, instrument administration and mock interviews. Except for web-based surveys in the USA, interviewers conducted face-to-face, tablet-assisted interviews in private households. For sensitive questions (eg, about recent sexual activity), respondents had the option to take the tablet and enter their responses privately.

Before beginning the DCE tasks, respondents received a standardised introduction explaining that the products were new hormonal contraceptive methods with safety and effectiveness comparable to existing options (eg, pills, implants and injectables). It also stated that products prevent pregnancy through hormonal mechanisms, do not affect long-term fertility, do not protect against HIV or other sexually transmitted infections, and would be available through common healthcare channels (online supplemental appendix S3).

To familiarise respondents with the seven hormonal product formats, all participants were shown seven fixed profiles—each representing a product profile defined by the same six attributes used in the DCE, with each attribute set at the most likely level under development for each product format (online supplemental appendix S4 and figures S2-S8). Each profile included visuals illustrating the product, its mode of administration, and the typical site of administration. The order of the seven profiles was randomised. After viewing each profile, respondents rated how likely they would be to use the product on a 5-point Likert scale; these data were not analysed in the current manuscript. A reference sheet displaying visuals of all product formats remained available for respondents to consult as needed during the DCE surveys.

Participants were also asked about demographic characteristics, sexual behaviour, fertility intentions and current contraceptive use before completing the DCE tasks. The survey took approximately 35 min to complete on average.

Interviewers entered all responses directly into a secure database in real time using the SurveyToGo platform (Dooblo Ltd., Kefar Sava).^27^ All country teams followed a harmonised data quality assurance protocol. Supervisors and data managers conducted daily checks for data consistency, including recruitment targets by interviewer and primary sampling units (eg, enumeration area), interview completeness, length and timing, Global Positioning System (GPS) coordinates, duplicate cases and audio uploads. In parallel, an independent team not affiliated with fieldwork teams conducted daily quality control using similar operational indicators, interview-level quality metrics and comparisons of overall sample characteristics against population benchmarks (eg, age distributions and prevalence of common contraceptive methods). For the DCE surveys, we evaluated completion of all 10 choice tasks, mean completion time and the proportions of respondents who consistently chose the left-hand option (Product X), the right-hand option (Product Y) or the ‘neither’ option across all 10 choice tasks.

### Statistical analysis

Baseline characteristics of study participants were summarised overall and by country using proportions for categorical variables and medians and IQRs for continuous variables. Information on current contraceptive method was collected only among non-pregnant women.

To estimate relative preference weights (i.e., utilities) for attributes, we fitted mixed logit models separately by each country. Since dosing intervals and time to return to fertility were tied to contraceptive format in the DCE design, we included format-specific interaction terms for dosing intervals in the models.

All attributes and interaction terms were effects-coded to ensure parameter estimates were relative to the overall mean utility rather than to a single reference level within each attribute.

Contraceptive format preferences were coded as random effects, as we hypothesised substantial between-individual preference heterogeneity, and because format was tightly linked to specific levels of other attributes. For the primary analyses, all other attribute coefficients were modelled as fixed effects to facilitate stable estimation and ease-of-interpretation of mean preferences.

The final model of the utility *U* for individual *n* choosing contraceptive alternative *j* in a choice task *t* was specified as:


$$U_{njt}=\;\sum_{k=1}^{K}\beta_{nk}x_{kjt}+\sum_{m=1}^{M}\beta_{m}z_{mjt}+\;\sum_{l=1}^{L}\beta_{l}w_{ljt}+\;\varepsilon_{njt}$$

where $x_{kjt}$ are effects-coded indicators for contraceptive format levels (k=1,…,K), $z_{mjt}$ are effects-coded indicators for all other attributes (m=1,…,M), and $w_{ljt}$ are interaction terms between contraceptive format and dosing interval (l=1,…,L)). The coefficients $\beta_{nk}$ are individual-specific (i.e., random) parameters capturing preference heterogeneity for contraceptive format, while $\beta_{m}$ and $\beta_{l}$ are fixed coefficients for other attributes and interaction terms. The term $\varepsilon_{njt}$ represents a stochastic error term.

Relative importance of each attribute was calculated using the range method,^28^ which measures the difference between the highest and lowest estimated coefficients across the levels of a given attribute and calculates it as a proportion of the total range summed across all attributes, scaled from 0% to 100%.

Our primary outcome was the set of relative preference weights (*β*), representing the marginal utility of each attribute level within the given attribute. More positive values indicate stronger preference (ie, greater utility) relative to the average preference across all levels within each attribute.

Models were estimated using the *logitr* package in R. Model estimation and reporting followed the International Society for Pharmacoeconomics and Outcomes Research (ISPOR) Good Research Practices for Conjoint Analysis, which sets international standards for the design and analysis of discrete choice experiments (Table S2).

### Sensitivity analysis

We fitted an additional model in which two additional attributes—effect on menstruation and weight—were specified as random coefficients, as these attributes showed substantial variation in their relative importance and preference weights across countries. This specification allowed us to capture individual-level preference heterogeneity and to assess whether allowing more attributes to vary randomly significantly altered the estimated preference weights.

### Informed consent

Participation in the survey was completely voluntary. Individuals could choose not to answer any question, decline participation in any component or withdraw at any time. Written informed consent was obtained from participants aged ≥18 years, and parental or guardian consent with child assent for those aged 15–17 years.

## Results

### Participant characteristics

A total of 20 068 participants in seven countries completed the DCE—approximately three thousand per country (table 2). DCE data from all respondents were included in the final analysis. Median age was 29 years (IQR: 24–35) and similar across countries. Regarding sexual frequency, 57% reported having sex at least once per week, and 31% at least once per month. Regarding number of sexual partners in the past 12 months—excluding Pakistan, where this question was not asked due to cultural sensitivity—8% of participants reported multiple partners, with the largest proportion in the USA (18%).

**Table 2:** Baseline characteristics of study participants by country

Characteristic	Overall n=20 068	Burkina Faso n=3027	Nigeria n=3051	Ethiopia n=2016	Kenya n=3059	Zambia n=2932	Pakistan n=2932	USA n=3051
Age, years								
Median (IQR)	29 (24, 35)	28 (22, 34)	29 (24, 35)	28 (23, 32)	28 (22, 35)	27 (22, 34)	30 (25, 35)	34 (28, 39)
Range, % (n)								
15–24	28% (5567)	35% (1057)	26% (786)	30% (611)	35% (1057)	40% (1163)	19% (556)	11% (337)
25–34	43% (8669)	41% (1227)	49% (1485)	49% (980)	38% (1158)	38% (1100)	49% (1423)	42% (1296)
35+	29% (5832)	25% (743)	26% (780)	21% (425)	28% (844)	23% (669)	33% (953)	46% (1418)
Education, % (n)								
No schooling	15% (2998)	40% (1202)	10% (314)	14% (288)	2.4% (72)	4.4% (129)	27% (791)	0% (0)
Primary‡	33% (6612)	31% (945)	10% (315)	37% (742)	34% (1030)	42% (1238)	31% (910)	7% (202)
Secondary	39% (7886)	25% (762)	43% (1323)	28% (564)	45% (1391)	48% (1396)	35% (1033)	16% (493)
Tertiary	13% (2572)	3.9% (118)	36% (1099)	21% (422)	19% (566)	5.8% (169)	6.8% (198)	77% (2356)
Marital status, % (n)								
Married or living together	77% (13 153)	86% (2614)	75% (2287)	93% (1878)	70% (2141)	70% (2060)	100% (2932)	71% (2173)
Divorced, separated or widowed	5.0% (858)	1.8% (53)	2.4% (74)	2.5% (51)	7.5% (228)	7.8% (230)	0% (0)	7.3% (222)
Never married	18% (3125)	12% (360)	23% (690)	4.3% (87)	23% (690)	22% (642)	0% (0)	22% (656)
Currently pregnant, % (n)								
Yes	9.2% (1837)	9.8% (298)	7.5% (229)	9.8% (197)	6.8% (209)	9.9% (289)	14% (422)	6.3% (193)
No	89% (17 900)	89% (2700)	90% (2757)	89% (1792)	92% (2809)	89% (2606)	85% (2486)	90% (2750)
Not sure	1.6% (331)	1.0% (29)	2.1% (65)	1.3% (27)	1.3% (41)	1.3% (37)	0.8% (24)	3.5% (108)
Current contraceptive method, % (n)**								
Daily pill	12% (2188)	8.5% (232)	11% (319)	9.1% (166)	8.4% (239)	12% (327)	9.2% (231)	24% (674)
Injection	19% (3448)	15% (418)	12% (350)	30% (539)	23% (665)	41% (1,073)	13% (326)	2.7% (77)
Implant	12% (2142)	23% (628)	7.3% (205)	22% (397)	19% (550)	8.1% (215)	1.5% (37)	3.8% (110)
Intrauterine device (IUD)	3.8% (696)	3.0% (83)	0.7% (20)	1.8% (33)	3.3% (94)	0.5% (13)	2.4% (60)	14% (393)
Vaginal ring	0.5% (98)	0.1% (3)	0.6% (18)	0.1% (2)	<0.1% (2)	0.2% (4)	1.0% (26)	1.5% (43)
Emergency contraceptive pill	1.0% (184)	<0.1% (2)	2.3% (66)	1.3% (24)	1.4% (40)	0.4% (11)	0.4% (11)	1.0% (30)
Condoms	14% (2595)	5.9% (161)	21% (584)	0.7% (13)	11% (304)	11% (292)	29% (730)	18% (511)
Withdrawal	5.8% (1062)	0.1% (3)	9.0% (254)	0.4% (8)	1.1% (31)	1.2% (32)	14% (354)	13% (380)
Rhythm method	3.2% (587)	3.0% (82)	4.1% (117)	6.3% (115)	3.8% (108)	1.0% (26)	0.7% (17)	4.3% (122)
Other methods†	2.3% (426)	0.8% (22)	3.2% (90)	4.5% (81)	0.9% (27)	2.3% (60)	2.4% (61)	3.0% (85)
None	26% (4805)	40% (1095)	28% (799)	24% (441)	28% (790)	22% (590)	26% (657)	15% (433)
Fertility intentions, % (n)								
Have another child	64% (12 772)	81% (2459)	76% (2312)	79% (1598)	60% (1838)	72% (2100)	46% (1352)	36% (1113)
No more	27% (5517)	9.4% (286)	18% (540)	16% (327)	35% (1070)	21% (629)	48% (1400)	41% (1265)
Undecided/don’t know	8.9% (1779)	9.3% (282)	6.5% (199)	4.5% (91)	4.9% (151)	6.9% (203)	6.1% (180)	22% (673)
Preferred timing for next child, % (n)								
<1 year	16% (2090)	18% (443)	20% (458)	18% (292)	10% (192)	13% (275)	8.1% (110)	29% (320)
1–2 years	25% (3197)	18% (433)	37% (846)	15% (245)	18% (322)	17% (364)	39% (534)	41% (453)
2–5 years	36% (4619)	45% (1104)	28% (637)	48% (766)	32% (592)	33% (702)	43% (576)	22% (242)
5+ years	16% (2100)	8.6% (212)	10% (235)	16% (249)	34% (633)	29% (611)	7.6% (103)	5.1% (57)
Don’t know	6.0% (766)	11% (267)	5.9% (136)	2.9% (46)	5.4% (99)	7.0% (148)	2.1% (29)	3.7% (41)
Number of sexual partners in past 12 months, % (n)								
1 partner	92% (15 753)	97% (2935)	92% (2804)	99% (1992)	92% (2805)	92% (2712)	Not applicable	82% (2505)
2+ partners	8.1% (1383)	3.0% (92)	8.1% (247)	1.2% (24)	8.3% (254)	7.5% (220)		18% (546)
Frequency of sex, % (n)								
At least once a week	57% (11 450)	49% (1480)	60% (1820)	63% (1273)	50% (1519)	61% (1791)	66% (1921)	54% (1646)
At least once a month	31% (6269)	36% (1103)	30% (914)	30% (606)	30% (918)	29% (847)	30% (892)	32% (989)
Less than once a month	12% (2349)	15% (444)	10% (317)	6.8% (137)	20% (622)	10% (294)	4.1% (119)	14% (416)

* Current contraceptive method information was collected among non-pregnant women only.

† Other methods include breastfeeding to stop period, vasectomy and other traditional method.

‡ For participants in the USA, the “Primary” category corresponds to less than high school education.

Fertility intentions and preferred timing for a next child were heterogeneous, with African countries having the highest proportion of participants desiring another child (60%–81%), and Pakistan and the USA having the highest proportion of participants desiring no more children (48%, 41%). Of those desiring another child, participants in the USA were most likely to desire having a child within the next year (29%), while participants in Kenya and Zambia were more likely to desire deferring the birth of a next child for 5 or more years (34% in Kenya, 29% in Zambia).

Current contraceptive method use varied widely across countries. Injectables were the most common method among participants in Zambia (41%), Kenya (23%) and Ethiopia (30%). Condoms were most common in Pakistan (29%) and Nigeria (21%). Implants were most common in Burkina Faso (23%), and daily pills in the USA (24%). Overall, 26% of participants reported not using any contraceptive method.

The mean completion time for the DCE was 8.0 (±11.1) minutes. Nearly all participants completed all 10 DCE choice tasks, with only four individuals (<0.1%) completing fewer than 10 tasks. Indicators of potentially non-differentiating response patterns were uncommon: 0.4% (74/20 068) always chose the left-hand option, and 0.1% (23/20 068) always chose the right-hand option. Overall, 11.4% (2287/20 068) always chose the ‘neither’ option across all tasks.

### Relative importance of six attributes

**Figure 1:**
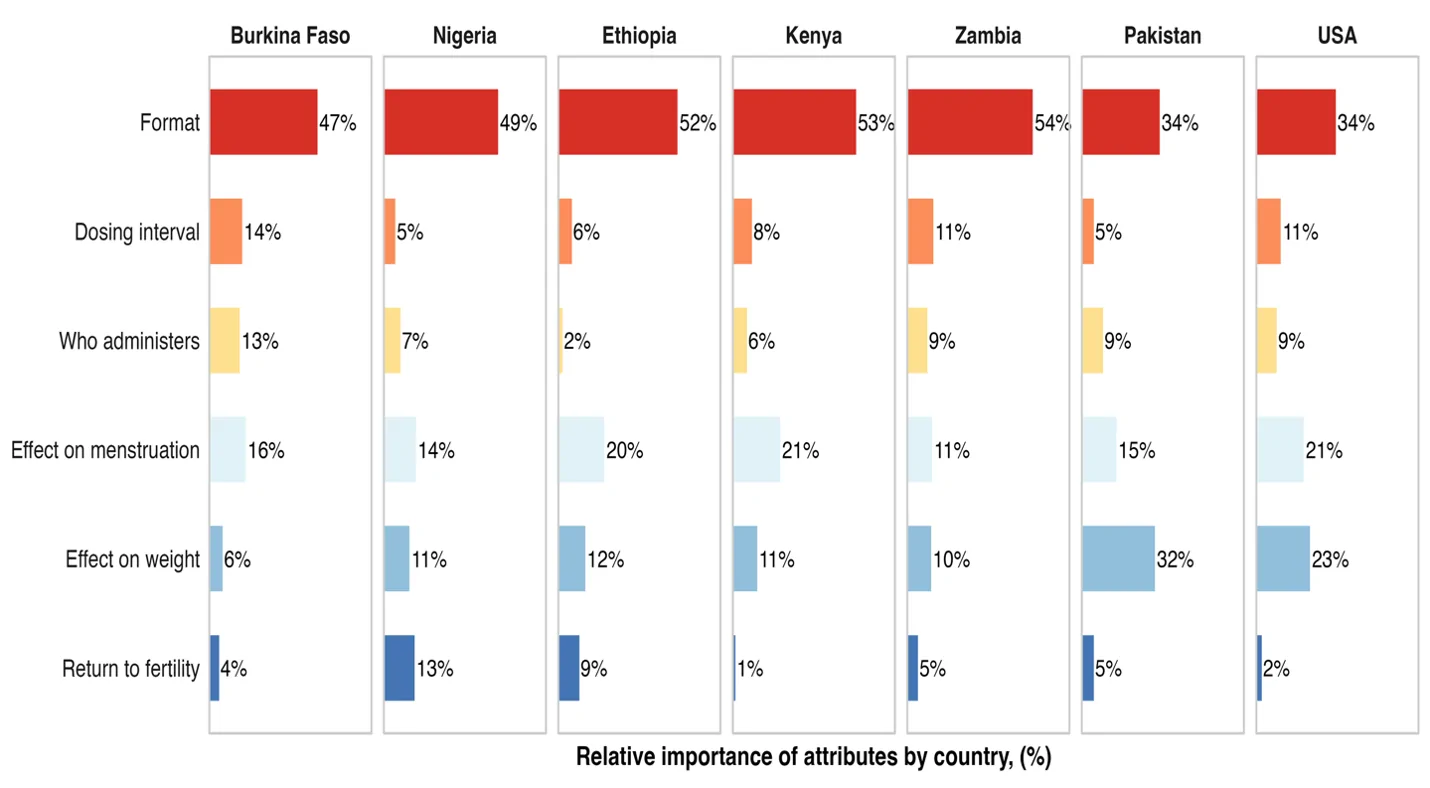
Relative importance of attributes by country.

Across all seven countries, format consistently emerged as the most important attribute, accounting for roughly one-third to more than half of the relative importance (34% in Pakistan and the USA to over 50% in Ethiopia, Kenya and Zambia) (figure 1). Effect on menstruation was the second most important attribute in Burkina Faso (16%), Nigeria (14%), Ethiopia (20%) and Kenya (21%), whereas effect on weight was more important in Pakistan (32%) and the USA (23%). Dosing interval, who administers, and time to return to fertility generally contributed less than 15% in all countries.

### Preference weights for contraceptive formats

**Figure 2:**
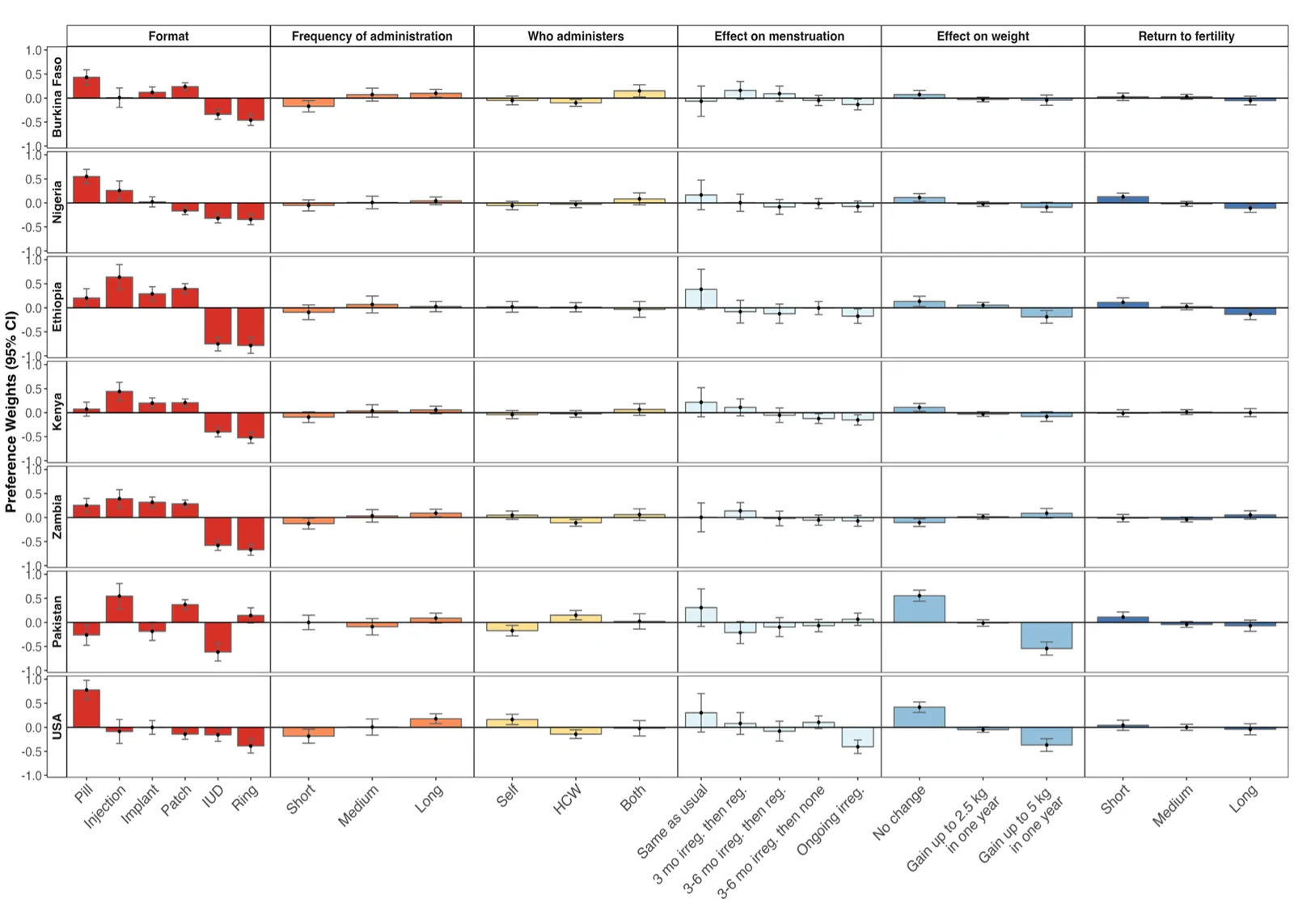
Preference weights for six contraceptive attributes by country. Bars represent estimated preference weights, and the error bars indicate 95% CIs. Positive values reflect stronger preference, while negative values reflect weaker preference compared with the average preference across all levels in a given attribute.

Contraceptive format consistently emerged as the most important attribute driving preferences (figure 2). However, random effects from the mixed logit models (table 3) revealed substantial heterogeneity in preferences for method format, suggesting diverse preference patterns across countries.

**Table 3:** Preference weights from mixed effects models in seven countries

Coefficients	Burkina Faso	Nigeria	Ethiopia	Kenya	Zambia	Pakistan	USA
Opt-out	0.18 (0.1, 0.25)***	0.37 (0.31, 0.44)***	0.19 (0.1, 0.28)***	0.5 (0.43, 0.57)***	0.04 (−0.03, 0.10)	1.96 (1.86, 2.07)***	0.5 (0.42, 0.58)***
Format							
Pill	0.43 (0.27, 0.59)***	0.55 (0.40, 0.70)***	0.2 (0.01, 0.40)*	0.07 (−0.07, 0.22)	0.26 (0.11, 0.4)***	−0.26 (−0.48, −0.05)*	0.78 (0.58, 0.98)***
Injection	0.01 (−0.19, 0.21)	0.26 (0.06, 0.46)*	0.64 (0.38, 0.90)***	0.44 (0.25, 0.63)***	0.39 (0.2, 0.58)***	0.55 (0.29, 0.81)***	−0.09 (−0.33, 0.16)
Implant	0.12 (0.01, 0.23)*	0.02 (−0.08, 0.13)	0.29 (0.15, 0.44)***	0.2 (0.09, 0.31)***	0.32 (0.21, 0.43)***	−0.18 (−0.38, 0)	0 (−0.14, 0.14)
Patch	0.24 (0.16, 0.32)***	−0.17 (−0.24, −0.09)***	0.4 (0.30, 0.50)***	0.21 (0.13, 0.28)***	0.29 (0.21, 0.36)***	0.37 (0.27, 0.47)***	−0.14 (−0.25, −0.04)**
IUD	−0.34 (−0.44, −0.23)***	−0.32 (−0.42, −0.22)***	−0.75 (−0.90, −0.61)***	−0.40 (−0.50, −0.30)***	−0.58 (−0.69, −0.47)***	−0.62 (−0.81, −0.43)***	−0.16 (−0.29, −0.02)*
Ring	−0.46 (−0.57, −0.35)***	−0.34 (−0.45, −0.24)***	−0.78 (−0.95, −0.62)***	−0.52 (−0.64, −0.41)***	−0.67 (−0.79, −0.56)***	0.15 (−0.01, 0.30)	−0.39 (−0.54, −0.24)***
Dosing interval							
Short	−0.17 (−0.29, −0.05)**	−0.05 (−0.17, 0.06)	−0.09 (−0.25, 0.06)	−0.09 (−0.21, 0.02)	−0.13 (−0.24, −0.01)*	0 (−0.15, 0.15)	−0.18 (−0.33, −0.04)*
Medium	0.07 (−0.06, 0.21)	0.01 (−0.12, 0.14)	0.07 (−0.11, 0.24)	0.04 (−0.09, 0.17)	0.03 (−0.10, 0.16)	−0.09 (−0.26, 0.08)	0.01 (−0.16, 0.17)
Long	0.1 (0.02, 0.18)*	0.04 (−0.04, 0.12)	0.03 (−0.08, 0.13)	0.06 (−0.02, 0.14)	0.09 (0.01, 0.17)*	0.09 (−0.01, 0.19)	0.18 (0.08, 0.28)***
Who administers							
Self	−0.05 (−0.14, 0.04)	−0.05 (−0.14, 0.03)	0.02 (−0.09, 0.13)	−0.04 (−0.13, 0.05)	0.05 (−0.04, 0.14)	−0.17 (−0.28, −0.06)**	0.16 (0.05, 0.27)**
HCW	−0.1 (−0.17, −0.03)**	−0.03 (−0.10, 0.04)	0.01 (−0.08, 0.11)	−0.03 (−0.1, 0.05)	−0.11 (−0.18, −0.04)**	0.15 (0.05, 0.25)**	−0.14 (−0.23, −0.05)**
HCW or self	0.15 (0.02, 0.28)*	0.08 (−0.04, 0.21)	−0.03 (−0.20, 0.13)	0.07 (−0.05, 0.19)	0.06 (−0.06, 0.18)	0.02 (−0.14, 0.18)	−0.02 (−0.18, 0.14)
Effect on menstruation							
Same as usual	−0.07 (−0.38, 0.25)	0.17 (−0.14, 0.47)	0.38 (−0.03, 0.80)	0.22 (−0.09, 0.52)	0 (−0.3, 0.31)	0.31 (−0.08, 0.70)	0.30 (−0.10, 0.70)
3 months irregular, then 1 week regular every month	0.16 (−0.02, 0.35)	0 (−0.17, 0.18)	−0.08 (−0.32, 0.16)	0.11 (−0.07, 0.29)	0.14 (−0.04, 0.31)	−0.21 (−0.44, 0.02)	0.08 (−0.15, 0.31)
3–6 months irregular, then 1 week regular every month	0.09 (−0.07, 0.25)	−0.08 (−0.24, 0.07)	−0.12 (−0.33, 0.08)	−0.05 (−0.20, 0.10)	−0.02 (−0.18, 0.13)	−0.1 (−0.29, 0.1)	−0.08 (−0.29, 0.13)
3–6 months irregular, then none	−0.05 (−0.16, 0.05)	−0.01 (−0.12, 0.09)	0 (−0.14, 0.13)	−0.12 (−0.23, −0.02)*	−0.05 (−0.16, 0.05)	−0.07 (−0.20, 0.06)	0.10 (−0.03, 0.23)
Ongoing irregular	−0.14 (−0.25, −0.03)*	−0.07 (−0.19, 0.04)	−0.17 (−0.32, −0.02)*	−0.15 (−0.26, −0.04)**	−0.07 (−0.18, 0.04)	0.07 (−0.06, 0.19)	−0.4 (−0.55, −0.26)***
Effect on weight							
No change	0.07 (−0.01, 0.16)	0.11 (0.03, 0.19)**	0.13 (0.03, 0.24)*	0.11 (0.03, 0.19)**	−0.11 (−0.19, −0.02)**	0.56 (0.44, 0.67)***	0.42 (0.31, 0.53)***
Gain up to 2.5 kg in 1 year	−0.03 (−0.08, 0.02)	−0.02 (−0.07, 0.03)	0.05 (0, 0.11)	−0.03 (−0.08, 0.02)	0.02 (−0.03, 0.07)	−0.01 (−0.08, 0.05)	−0.05 (−0.1, 0.01)
Gain up to 4.5 kg in 1 year	−0.04 (−0.15, 0.06)	−0.09 (−0.19, 0.01)	−0.19 (−0.32, −0.05)**	−0.08 (−0.18, 0.02)	0.09 (−0.01, 0.19)	−0.54 (−0.68, −0.41)***	−0.37 (−0.50, −0.24)***
Time from discontinuation to return to fertility							
Short	0.03 (−0.05, 0.10)	0.13 (0.05, 0.20)***	0.11 (0.02, 0.21)*	−0.01 (−0.08, 0.06)	−0.01 (−0.09, 0.06)	0.11 (0, 0.22)*	0.04 (−0.06, 0.15)
Medium	0.03 (−0.03, 0.08)	−0.02 (−0.07, 0.03)	0.02 (−0.04, 0.09)	0.01 (−0.04, 0.06)	−0.04 (−0.09, 0.01)	−0.04 (−0.10, 0.02)	0 (−0.06, 0.06)
Long	−0.05 (−0.14, 0.04)	−0.11 (−0.20, −0.02)*	−0.14 (−0.25, −0.02)*	0 (−0.08, 0.09)	0.05 (−0.03, 0.14)	−0.07 (−0.19, 0.05)	−0.04 (−0.15, 0.07)
Random effects coefficients (SD)							
Injection	−0.67 (−0.76, −0.59)***	−0.63 (−0.71, −0.55)***	−0.6 (−0.71, −0.50)***	−0.67 (−0.75, −0.58)***	−0.72 (−0.8, −0.64)***	0.83 (0.67, 0.98)***	1.13 (1.03, 1.23)***
Implant	−0.82 (−0.91, −0.72)***	−0.75 (−0.83, −0.67)***	0.72 (0.62, 0.81)***	0.73 (0.66, 0.81)***	0.67 (0.59, 0.75)***	−0.91 (−1.11, −0.70)***	−1.27 (−1.38, −1.16)***
Patch	0.85 (0.79, 0.91)***	−0.82 (−0.89, −0.75)***	0.85 (0.77, 0.93)***	0.80 (0.73, 0.86)***	0.85 (0.79, 0.92)***	1.22 (1.1, 1.34)***	−1.41 (−1.50, −1.31)***
IUD	−1.03 (−1.15, −0.91)***	−0.65 (−0.74, −0.56)***	−1.16 (−1.32, −1.00)***	−0.96 (−1.08, −0.85)***	−0.97 (−1.09, −0.86)***	−1.34 (−1.6, −1.09)***	−1.59 (−1.72, −1.44)***
Ring	0.67 (0.58, 0.76)***	0.69 (0.63, 0.76)***	0.99 (0.89, 1.09)***	0.72 (0.63, 0.80)***	0.77 (0.69, 0.85)***	0.55 (0.41, 0.69)***	1.56 (1.45, 1.67)***

*<0.05; **<0.01; ***< 0.001.

Interaction terms between format and dosing interval were included in the model but the results are not shown due to table length. Full results, including all interaction terms, are presented in online supplemental table S3.

HCW, healthcare worker; IUD, intrauterine device.

Injectable formats had positive preference weights in all countries except the USA, where the direction of association was negative but not statistically significant. The strongest positive preference weights for injectable contraceptives were observed in Ethiopia (β=0.64; 95% CI 0.38 to 0.90), followed by Pakistan (β=0.55; 95% CI 0.29 to 0.81), Kenya (β=0.44; 95% CI 0.25 to 0.63) and Zambia (β=0.39; 95% CI 0.20 to 0.58).

Oral pills had positive preference weights in all countries except for Pakistan and Kenya. The largest positive preference weight for pills was observed in the USA (β=0.78, 95% CI 0.58 to 0.98). Other countries also showed moderate positive preference weights favouring pills, including Burkina Faso (β=0.43; 95% CI 0.27 to 0.59) and Nigeria (β=0.55; 95% CI 0.40 to 0.70).

IUDs and vaginal rings had negative preference weights in all countries except in Pakistan, where the direction of preference for the vaginal ring coefficient was not statistically significant. The strongest negative preference weights were observed for the IUD in Ethiopia (β=−0.75; 95% CI −0.90 to −0.61) and Pakistan (β=−0.62; 95% CI −0.81 to −0.43), and for the vaginal ring in Ethiopia (β=−0.78; 95% CI −0.95 to −0.62]) and Zambia (β=−0.67; 95% CI −0.79 to −0.56).

Preference weights for implants and micro-array patches varied across countries. Implants had statistically significant positive preference weights in Ethiopia, Kenya and Zambia, but not in the other countries. Micro-array patches were associated with positive preference weights in Burkina Faso, Ethiopia, Kenya, Zambia and Pakistan, but not in Nigeria and the USA.

### Preference weights for contraceptive attributes other than format

Avoiding weight gain was associated with positive preference weights in several countries, including Nigeria (β=0.11; 95% CI 0.03 to 0.19), Ethiopia (β=0.13; 95% CI 0.03 to 0.24) and Kenya (β=0.11; 95% CI 0.03 to 0.19), and most strongly in Pakistan (β=0.56; 95% CI 0.44 to 0.67) and the USA (β=0.42; 95% CI 0.31 to 0.53). Conversely, a weight gain of up to 4.5 kg was significantly associated with negative preference weights in Pakistan (β=−0.54; 95% CI −0.68 to −0.41) and the USA (β=−0.37; 95% CI −0.50 to −0.24), with smaller or non-significant effects in other countries (figure 2 and table 3).

Across countries, there was a consistent directional trend toward lower preference weights for methods that caused ongoing irregular bleeding (figure 2). Ongoing irregular bleeding was associated with negative preference weights in the USA (β=−0.40; 95% CI −0.55 to −0.26), Kenya (β=−0.15; 95% CI −0.26 to −0.04) and Burkina Faso (β=−0.14; 95% CI −0.25 to −0.03), with similar but non-significant negative trends in some other settings.

Preference weights for contraceptive self-administration were generally not statistically significant. Contraceptives that could only be provider-administered had weak but statistically significant negative preference weights in Burkina Faso (β=−0.10; 95% CI −0.17 to −0.03), Zambia (β=−0.11; 95% CI −0.18 to −0.04) and the USA (β=−0.14; 95% CI −0.23 to −0.05), whereas a positive preference weight was observed in Pakistan (β=0.15; 95% CI 0.05 to 0.25). Contraceptives with flexibility to be either self-administered or provider-administered had negative preference weights in Burkina Faso, with no statistically significant direction of preference in other countries.

Time from discontinuation until return-to-fertility and dosing interval also had smaller magnitudes of preference weights compared with other attributes, but showed consistent patterns across countries. Across all countries, there was a trend toward positive preference weights for faster return-to-fertility and less frequent administration. For example, the least frequent administration (longest duration) was associated with small but statistically significant positive preference weights in Burkina Faso (β=0.10; 95% CI 0.02 to 0.18) and the USA (β=0.18; 95% CI 0.08 to 0.28).

When exploring the interaction between format and dosing interval, we observed trends toward preferring longer-acting methods for all formats except for the micro-array patch (online supplemental figure S9). There was a trend toward preferring shorter-acting patches in Pakistan, Zambia, Nigeria and Burkina Faso, and a trend toward preferring longer-acting patches in the USA, Ethiopia and Kenya. However, these interaction effects were small in magnitude and often estimated with substantial uncertainties, and many did not reach thresholds for statistical significance.

### Sensitivity analyses

The model that specified effect on menstruation and weight as random coefficients in addition to format yielded similar qualitative results that format remained the dominant driver of preferences, and the relative ranking of effect on menstruation and weight was consistent with the main models. Estimates from these sensitivity analyses are presented in the online supplemental table S4 and figure S10.

## Discussion

In the largest multi-country study to date quantifying preferences for contraceptive attributes, format was the most influential attribute. Nearly half of the total preference weights were attributable to the product format, yet format preferences were heterogeneous within and across countries. Injectables were preferred in Kenya, Ethiopia, Zambia and Pakistan, while pills were preferred in Burkina Faso, Nigeria and the USA. In all countries except the USA and Pakistan, IUDs and vaginal rings were strongly less preferred. Except for in Nigeria and the USA, micro-array patches were preferred, although less strongly than some other formats. After contraceptive format, effect on weight was the second most influential attribute in Pakistan and USA, whereas effect on menstrual patterns was more important in the other countries. In both the USA and Pakistan, weight gain held a similar relative importance to format. Dosing interval, when varied over a format-appropriate range, was generally of lower importance. Longer-acting options were generally preferred except in the case of micro-array patches, where trends were mixed.

Our findings complement existing literature on the importance of familiarity, usability and perceived ease-of-use in contraceptive decision-making. Prior studies identified a lack of familiarity and fear of insertion-related discomfort as major barriers to uptake, particularly for IUDs,^29^ as well as concerns about IUDs and vaginal rings being dislodged during sex or other activities.^29^ A review of acceptability trials found that these concerns and lack of familiarity with IUDs and vaginal rings were a barrier to continuation, even in the presence of favourable side-effect profiles.^30 31^ Stated preferences in our DCE likely reflect both underlying preferences and learnt familiarity based on prior exposure and available methods in each context. For example, strong preferences for injectables in Ethiopia and Kenya and for pills in the USA, Burkina Faso and Nigeria likely reflect cumulative experience, familiarity and programmatic emphasis on these methods. In contrast, we found that micro-array patches—a relatively novel format—showed moderate preference in most countries except the USA and Nigeria. These patterns suggest potential for greater acceptability of newer formats with increased awareness, real-world availability and product familiarity for novel formats. Our estimates should be interpreted as a snapshot of preferences under current conditions, rather than stable, long-term valuations.

Our findings provide insights for contraceptive product development and delivery. The importance of contraceptive format suggests that while improving side effect profiles is valuable, innovations focused solely on side effects may be insufficient if they are not offered in a format that aligns with users’ preferences. Format innovation should be accompanied by user-centred design and strategies to improve familiarity and acceptability. Evidence from this study can support rights-based, choice-driven contraceptive programming, emphasising the need for a range of accessible options tailored to the needs and preferences of specific populations.

Despite this study constituting what we believe to be the largest of its kind, many of the observed trends in preference weights did not reach statistical significance, suggesting opportunities for further research. This may reflect both the dominance of format in driving preferences and that preference weights for some attributes are small in magnitude, which limits statistical power and precision to detect modest differences in preferences. For example, while there was a trend toward preferring less frequent administration in all countries, it only reached statistical significance in Burkina Faso, Zambia and the USA. To robustly understand secondary factors influencing preferences for contraceptive options, it may be necessary to focus on a narrower set of product formats and attributes. Such studies may consider using a shared, interoperable DCE in order to facilitate data pooling for broader, multi-product, multi-country insights such as those presented here.

The current analysis focused on national-level estimates by country; however, preferences may vary by sociodemographic characteristics such as age, parity, fertility intentions or current method of use. Detailed subgroup analyses were beyond the scope of this cross-country analysis. We plan to conduct secondary analyses to examine preference heterogeneity and provide more targeted insights for programme design and strategies that better reflect the diversity of users’ needs and preferences within countries.

This study has several limitations. First, we did not include attributes related to cost. Contraception is often subsidised due to its high value to society and health systems, making end-user cost a question that is highly intertwined with health financing policy and beyond the scope of the current study. Second, we excluded multi-purpose prevention technologies (eg, combined HIV/STI and pregnancy prevention), which are under active development and align with stated user preferences in many LMICs.^32 33^ Third, while the DCE was designed to ensure that only plausible attribute combinations were considered, this necessarily limited full independence between attributes and may have constrained the exploration of hypothetical ideal combinations. Also, due to this structured dependence, estimates for dosing interval and return-to-fertility reflect preferences over plausible ranges within each format. It is also possible that format served as a proxy not only for delivery route but also for bundles of expectations about dosing intervals and return-to-fertility timing, even though these dimensions were modelled explicitly. Fourth, we did not include repeated choice tasks that could have been used for internal consistency checks. However, estimated attribute preference weights were generally in the expected direction (eg, no weight change was preferred over weight gain), suggesting overall coherence in respondents’ stated preferences. Finally, though our study spanned seven countries across three continents and the full spectrum of country income groups, important global regions including Europe, Eastern Asia and Central and South America were missing from our study sample, limiting generalisability. Although the DCE design, attribute set and analytic models were standardised across countries, differences in sampling frames, survey modes, baseline familiarity with hormonal methods and broader country-specific contexts may limit the extent to which quantitative preference weights can be compared directly across countries. Our primary focus is therefore on within-country patterns, and we use cross-country comparisons primarily to characterise the relative ranking and direction of preference weights rather than to make precise cross-country effect size comparisons.

However, strong representation from sub-Saharan Africa was a notable strength, given that this region faces the world’s highest rates of unintended pregnancy^23^ and maternal mortality,^24^ yet is often under-represented in biomedical research. Increasing access to preferred contraceptive methods is widely recognised as a key intervention to reduce unintended pregnancies and maternal mortality.^4^

In summary, findings from this multi-country study suggest that contraceptive format was the most influential factor shaping women’s preferences, outweighing attributes such as dosing interval, time to return to fertility, bleeding changes, weight gain and ability to self-administer. While preferences for other attributes were relatively consistent across countries—eg, preferring less frequent administration, avoiding weight gain and avoiding prolonged irregularities or increases in bleeding—format preferences were heterogeneous both within and across countries. National-level estimates may not fully capture heterogeneity across user subgroups, which will be explored further in future analyses. These findings may help inform developers, donors and policymakers seeking to design, fund and implement contraceptive technologies that are better aligned with end-user preferences.

### Patient and public involvement

Patients or the public were not involved in the design, or conduct, or reporting, or dissemination plans of this research.

## Supplementary material

10.1136/bmjgh-2025-023196 online supplemental file 1

10.1136/bmjgh-2025-023196 online supplemental file 2

## Data Availability

Data may be obtained from a third party and are not publicly available.
