# Supplementary material for: Preferences for characteristics of contraceptive methods: a large, multi-country discrete choice experiment
Source: BMJ Glob Health. 2026 Aug 24;11(8):e023196. doi: 10.1136/bmjgh-2025-023196 (PMC13504926; doi:10.1136/bmjgh-2025-023196)
Supplement: Supplementary file 1 [file bmjgh-11-8-s001.docx]

**Author Reflexivity Statement for**: Convergence of HIV and noncommunicable disease epidemics: Geospatial mapping of the unmet health needs in a HIV hyperendemic community in South Africa

Hae-Young Kim,^1^ Fred Church,^2^ LinChiat Chang,^3^ Della Berhanu,^4,5^ John Kinuthia,^6^ Anna Bershteyn^1^

^1^ Department of Population Health, New York University Grossman School of Medicine, New York, NY, USA

^2^ Format Analytics, Rockaway, NJ, USA

^3^ Independent Consultant, Cape Town, South Africa

^4^ Ethiopian Public Health Institute, Addis Ababa, Ethiopia

^5^ London School of Hygiene & Tropical Medicine, London, United Kingdom

^6^ Department of Research & Programs, Kenyatta National Hospital, Nairobi, Kenya

***1. How does this study address local research and policy priorities?***

This study directly addresses national priorities across all participating countries to reduce unintended pregnancies and improve access to modern contraceptive options. By generating nationally representative, census-guided estimates of women’s preferences for characteristics of hormonal contraceptives, this study provides empirical evidence to support procurement, guidelines, and method introduction strategies. The cross-country design also enables comparisons across policy environments in sub-Saharan Africa and South Asia, supporting regional and global stakeholders (e.g., donors, implementing partners, and product developers) in prioritizing products that align with locally expressed needs and preferences.

***2. How were local researchers involved in study design?***

Local researchers and research organizations from each participating country were engaged from formative phase through finalization of the study protocol. They contributed to: (i) adapting eligibility criteria to cultural norms (e.g., adjusting age eligibility and marital status criteria in Pakistan); (ii) reviewing and refining DCE attributes and translations to ensure that terminology, descriptions, and visual aids were locally understandable and culturally appropriate; (iii) advising on sampling strategies based on national census frameworks and urban–rural distributions; and (iv) leading piloting and cognitive pre-testing, which improved attribute comprehension, visual design, and interview flow. Local partners also led all in-country data collection, including interviewer recruitment and training, field supervision, and quality monitoring (e.g., GPS check, audio audits, and review of interview durations).

***3. How has funding been used to support the local research team?***

Gates Foundation funding (INV-022981) directly supported local research team. This included support for personnel costs for local investigators, project coordinators, and field supervisors; training and remuneration for interviewers; and operational expenses such as travel, tablet procurement and maintenance, and secure data transmission. In addition, a separate Gates Foundation funding (INV-085029) provided support for Drs. Berhanu and Kinuthia, enabling their contribution to their data interpretation and manuscript writing.

***4. How are research staff who conducted data collection acknowledged?***

Field partners and team leaders are acknowledged by name and institution in the Acknowledgements section (e.g., GVS Research in Kenya, Ethiopia, and Burkina Faso; MCDS Research in Nigeria; BrandComm Limited in Zambia; and Markematics in Pakistan). Individual field leads are acknowledged for their central roles in coordinating and overseeing data collection and ensuring data quality. We also acknowledge the broader field teams in each country for their support and contributions to data collection.

***5. Do all members of the research partnership have access to study data?***

All core local partner organizations involved in design and implementation have access to de-identified datasets.

***6. How was data used to develop analytical skills within the partnership?***

Local partner organizations were oriented to the full study protocol, including the structure of the DCE and the planned analytical approach. Although data collection partners were not expected to conduct formal analysis, the analytic team, which included Drs. Berhanu and Kinuthia, both practicing physicians and researchers in the field of sexual and reproductive health, met regularly to review methodological approaches, model selection, preliminary result, and interpretation. These meetings also provided opportunities to review country-level outputs and gather contextual insights and considerations based on clinical practice and programmatic experience. Dr. Chang played a key role in designing the DCE and contributed substantially to the analytical work.

***7. How were research partners supported to develop writing skills?***

We developed the manuscript through a collaborative and iterative process. Early drafts of the outline, tables, and figures were circulated to all co-authors, who were invited to contribute where relevant. Iterative rounds of revision were conducted through shared documents and regular group calls, during which all co-authors were encouraged to suggest alternative interpretations and refine the framing of the findings. The senior author provided detailed feedback and edits to strengthen scientific argumentation and clarity of presentation.

***8. How have research partners collaborated in interpreting study data?***

Interpretation of study findings incorporated the expertise of clinicians and researchers familiar with contraceptive provision and program implementation in the study settings. These insights helped contextualize the results in relation to current method mix, provider practices, and community norms. Feedback from these partners informed revisions to the Discussion and country-specific interpretations, ensuring that the findings accurately reflected local experiences and programmatic contexts.

***9. How will research products be shared to address local needs?***

Study findings will be disseminated through key stakeholder meetings co-organized with local partners, wherever feasible. Where appropriate, summaries will be presented at local technical working groups focused on reproductive health.

***10. How is the leadership, contribution, and ownership of this work by LMIC researchers recognized within the authorship?***

Authorship was determined according to publicly available ICMJE criteria. Local partners who contributed substantially to 1) the study design, data acquisition, or data interpretation; and 2) the review of analytical outputs were included as co-authors. All co-authors also reviewed and approved the manuscript. The Contributors section describes the specific roles of each author.

***11. How have early career researchers across the partnership been included within the authorship team?***

The lead author is an early career researcher, who led the data analyses, generated all figures and tables, and drafted the first version of the manuscript. Other early career researchers were also engaged throughout the project.

***12. How has gender balance been addressed within the authorship?***

The authorship team includes both women and men across career stages and institutions. Individuals of all genders who played substantive roles in study design, implementation, analysis, and manuscript development were recognized as co-authors.

***13. How has the project contributed to the training of LMIC researchers?***

The project contributed to the training opportunities for LMIC researchers through a combination of formal and informal capacity-building activities that were carried out collaboratively with local research leads. While the study team provided initial training such as administering the discrete choice experiment surveys and data collection using tablet-based platforms, much of the day-to-day training and skill development for the broader field teams was led by local team leaders. Local supervisors trained interviewers, coordinated field procedures, and oversaw real-time data quality monitoring, reflecting their existing expertise and strengthening capacity through collaborative practice. The skills developed through this partnership are transferable to future survey research and other studies within their institutions.

***14. How has the project contributed to improvements in local infrastructure?***

Funding supported the purchase and maintenance of data collection tablets, licenses for electronic survey platforms, and secure data storage solutions. Standard operating procedures (SOPs) for fieldwork, data security, and quality monitoring were developed collaboratively and can be reused or adapted for future studies. While the project did not involve physical laboratory infrastructure, it enhanced the digital and organizational infrastructure for high-quality, large-scale survey research in each participating country.

***15. What safeguarding procedures were used to protect local study participants and researchers?***

For participants, all interviews were conducted in private settings, and sensitive questions (e.g., sexual activity) could be answered directly on the tablet to minimize discomfort and risk of inadvertent disclosure. Participation was voluntary, with informed consent (and assent with parental/guardian consent for minors) obtained according to local ethical requirements. Participants were informed that they could skip any question or withdraw at any time without consequences. No identifying information was stored with DCE responses; data were encrypted and transmitted to secure servers, and all analyzed data were fully de-identified.

For research staff, interviewer training included modules on research ethics, confidentiality, and managing sensitive topics respectfully. Ethical approvals were obtained from national and institutional review boards in all participating countries, and the protocol complied with international standards for human subjects research, including strict adherence to data protection and confidentiality requirements.
