# Supplementary material for: Preferences for characteristics of contraceptive methods: a large, multi-country discrete choice experiment
Source: BMJ Glob Health. 2026 Aug 24;11(8):e023196. doi: 10.1136/bmjgh-2025-023196 (PMC13504926; doi:10.1136/bmjgh-2025-023196)
Supplement: Supplementary file 2 [file bmjgh-11-8-s002.docx]

**Supplementary Appendix for:**

**Preferences for characteristics of contraceptive methods: a large, multi-country discrete choice experiment**

Table of Contents

[Figure S1. Sample choice task of the discrete choice experiment	2](#_Toc224857401)

[S1. Sample Size Calculation	3](#_Toc224857402)

[S2. Sampling Strategy	3](#_Toc224857403)

[S3. Introduction Vignette for the DCE	4](#_Toc224857404)

[S4. Seven Fixed Profiles	5](#_Toc224857405)

[**Figure S2.** Visual illustration and profile card of the 6-month injectable method	6](#_Toc224857406)

[**Figure S3.** Visual illustration and profile card of the dissolving implant method	7](#_Toc224857407)

[**Figure S4.** Visual illustration and profile card of the micro-array patch method	8](#_Toc224857408)

[**Figure S5.** Visual illustration and profile card of the monthly pill method	9](#_Toc224857409)

[**Figure S6.** Visual illustration and profile card of the hormonal IUD method	10](#_Toc224857410)

[**Figure S7.** Visual illustration and profile card of the on-demand pill method	11](#_Toc224857411)

[**Figure S8.** Visual illustration and profile card of the vaginal ring method	12](#_Toc224857412)

[Table S1. Dosing interval and levels of time to return to fertility (RTF) by contraceptive format	13](#_Toc224857413)

[Table S2. Checklist for conjoint analysis applications in health care description	14](#_Toc224857414)

[Table S3. Preference weights from mixed effects models in seven countries	16](#_Toc224857415)

[Figure S9. Preference weights by contraceptive format and format-specific dosing interval.	18](#_Toc224857416)

[Table S4. Preference weights from mixed effects models across seven countries, with random effects for format, effect on menstruation, and effect on weight.	19](#_Toc224857417)

[Figure S10. Preference weights for six contraceptive attributes by country from mixed effects models with random effects for format, effect on menstruation, and effect on weight.	22](#_Toc224857418)

| **PRODUCT X** | **PRODUCT Y** | **NEITHER** |
| --- | --- | --- |
| **What is it?** | | **I do NOT want to use X or Y** |
| **V-ring** | **Micro-array Patch** |  |
| **How long does it last?** | |  |
| **1 year** | **3 months** |  |
| **Who can do it?** | |  |
| **You can do it yourself** | **You can do it yourself** |  |
| **Effect on period?** | |  |
| **Irregular bleeding for 3 to 6 months, then regular bleeding for 1 week every month** | **The same as your current bleeding** |  |
| **Effect on body weight?** | |  |
| **You may gain up to 2.5kg in one year** | **You may gain up to 4.5kg in one year** |  |
| **How soon can I get pregnant**  **after I stop using it?** | |  |
| **Within 1 month** | **Within 3 months** |  |

## **Figure S1**. Sample choice task of the discrete choice experiment

## **S1. Sample Size Calculation**

Using Orme’s rule-of-thumb,^[1],^^[2]^ the estimated minimum required sample size to obtain a representative estimate of contraceptive preference for a given group was $N\geq\frac{500*c}{t*a}$ with *c* representing the number of parameters estimated for an alternative, *t* representing the number of choice tasks per respondent, and *a* representing the number of alternatives per task.

In this study, each respondent completed *t*=10 choice tasks with *a*=3 alternatives per task. Each alternative included *c=*12 parameters: 10 main-effect estimates of preferences for contraceptive attributes, and 2 format-dosing interval interaction terms, which were added because contraceptive formats were assigned different relevant frequencies of administration.

This yielded a minimum sample size of $N\geq\frac{500*12}{10*3}=200$ per group, suggesting that national-level estimates could be obtained with as few as 200 respondents per country. However, we aimed to exceed this target due to high anticipated preference heterogeneity and to allow for future subnational mapping and subgroup analyses. We estimated that subnational analysis would require 10–15 geographic or sub-population segments, which would also enable more robust estimates in the national analysis. Therefore, we targeted 3,000 participants per country.

## **S2. Sampling Strategy**

We aimed to recruit a nationally representative sample where possible. In all countries except the US, we used national census data to implemented multi-stage random cluster sampling with probability proportional to size, stratified by urban vs. rural area classification to ensure geographic representativeness. This sampling strategy produced a set of census enumeration areas designated as the study’s primary sampling units (PSUs).

In five countries (Burkina Faso, Ethiopia, Kenya, Nigeria, and Zambia), 600 PSUs were randomly selected per country. In the US, due to its large size, we employed a hybrid sampling design with both probability-based and non-probability web panels; recruitment was calibrated to adjust for self-selection bias in the non-probability sample. In Ethiopia, only 400 PSUs were selected in the south of the country, as it was not safe for research teams to operate in the north of the country due to civil war.

Within each PSU, the study team identified eligible households, defined as occupied dwellings with at least one woman resident in the eligible age range. Of these, 10 households per PSU were selected by a random number draw. If more than one woman in the age range resided in the same household, one was randomly selected using the most recent birthday method (i.e., choosing one whose birthday was soonest after the interview date).

Because the DCE was embedded within a larger study, participants were randomly assigned to either complete the DCE reported here, or to complete different study activities, in a 1:1 ratio. This approach avoided excessive cognitive burden from completing multiple study activities. The analysis presented here only includes the half of study participants who answered the DCE survey questions.

## **S3. Introduction Vignette for the DCE**

All respondents were first introduced through the following standardized script read by the interviewer:

*“In this section, I will show you a few new methods to avoid pregnancy.*

*The ability of these products to prevent unwanted pregnancy is similar to existing contraceptive methods such as daily pills, implants and injectables.*

*The safety of these products is similar to existing contraceptive methods such as daily pills,*

*implants and injectables.*

*All of these products deliver hormones that prevent you from becoming pregnant.*

*The products do not cause abortion. If you were to become pregnant while using them, they*

*would not harm your baby.*

*The products have no long-term effects on your fertility. You will be able to get pregnant once you stop using them.*

*The products do not protect you from HIV or other sexually transmitted infections.*

*The products will be available from your local health care provider or health center, or from a pharmacy.”*

## **S4. Seven Fixed Profiles**

All respondents then viewed a series of contraceptive product profiles (X01–X07), each describing one of the new contraceptive methods. For each profile, respondents rated how likely they would be to use the product on a 5-point Likert scale. The order of presentation was randomized across respondents.

*“I am going to show you a new method to avoid pregnancy.”*

Each respondent was presented with a visual depiction of the method and a card illustrating its attributes and attribute levels (Figures S1-S7). After viewing the card, respondents were asked:

*“How likely are you to use this method?*

***Response options (five-point Likert scale):***

*1 = Definitely not
2 = Probably not
3 = Might or might not
4 = Probably
5 = Definitely”*

For the vaginal ring method only, respondents were provided with the following additional explanation:

*“Each month, the ring is inserted into your vagina for three weeks and removed for one week. A single ring can be used for one year.”*

**Figure S2.** Visual illustration and profile card of the 6-month injectable method

**Figure S3.** Visual illustration and profile card of the dissolving implant method

**Figure S4.** Visual illustration and profile card of the micro-array patch method

**Figure S5.** Visual illustration and profile card of the monthly pill method

**Figure S6.** Visual illustration and profile card of the hormonal IUD method

**Figure S7.** Visual illustration and profile card of the on-demand pill method

**Figure S8.** Visual illustration and profile card of the vaginal ring method

## **Table S1. Dosing interval and levels of time to return to fertility (RTF) by contraceptive format**

|  | **Injection** | **Implant** | **Micro-Array Patch** | **Monthly Pill** | **IUD** | **On-Demand Pill** | **Vaginal Ring** |
| --- | --- | --- | --- | --- | --- | --- | --- |
|  |  |  |  |  |  |  |  |
| Dosing interval: Short | 3 months | 6 months | 3 months | 24 hours before/after | 6 months | 24 hours before/after | 1 month |
| RTF A: months | 1 | 3 | 1 | 1 | 3 | 1 | 1 |
| RTF B: months | 3 | 6 | 3 | 2 | 6 | 2 | 2 |
| RTF C: months | 6 | 12 | 6 | 3 | 12 | 3 | 3 |
|  |  |  |  |  |  |  |  |
| Dosing interval: Medium | 4 months | 1 year | 4 months | 1 month | 2 years | 1 month | 3 months |
| RTF A: months | 1 | 1 | 1 | 1 | 6 | 1 | 1 |
| RTF B: months | 3 | 2 | 3 | 2 | 12 | 2 | 3 |
| RTF C: months | 6 | 3 | 6 | 3 | 18 | 3 | 6 |
|  |  |  |  |  |  |  |  |
| Dosing interval: Long | 6 months | 2 years | 6 months | 3 months | 5 years | 3 months | 1 year |
| RTF A: months | 3 | 6 | 3 | 1 | 1 | 1 | 1 |
| RTF B: months | 6 | 12 | 6 | 3 | 2 | 3 | 2 |
| RTF C: months | 12 | 18 | 12 | 6 | 3 | 6 | 3 |

## **Table S2. Checklist for conjoint analysis applications in health care description**

*Adapted from* Bridges JFP, Hauber AB, Marshall D, Lloyd A, Prosser LA, Regier DA, et al. Conjoint Analysis Applications in Health—a Checklist: A Report of the ISPOR Good Research Practices for Conjoint Analysis Task Force. Value in Health. 2011 June 1;14(4):403–13.

| **Checklist for conjoint analysis applications in health care description** | **Reported on sections & paragraphs (page)** |
| --- | --- |
| 1. Was a well-defined research question stated and is conjoint analysis an appropriate method for answering it? |  |
| 1.1 Were a well-defined research question and a testable hypothesis articulated? | Introduction (pg. 6-7) |
| 1.2 Was the study perspective described, and was the study placed in a particular decision-making or policy context? | Introduction (pg. 6-7) |
| 1.3 What is the rationale for using conjoint analysis to answer the research question? | Introduction (pg. 5-6) |
| 2. Was the choice of attributes and levels supported by evidence? |  |
| 2.1 Was attribute identification supported by evidence (literature reviews, focus groups, or other scientific methods)? | Methods, Selection of contraceptive product features for the choice experiment (pg. 7) |
| 2.2 Was attribute selection justified and consistent with theory? | Methods, Selection of contraceptive product features for the choice experiment (pg. 7) |
| 2.3 Was level selection for each attribute justified by the evidence and consistent with the study perspective and hypothesis? | Methods, Selection of contraceptive product features for the choice experiment (pg. 7) |
| 3. Was the construction of tasks appropriate? |  |
| 3.1 Was the number of attributes in each conjoint task justified (that is, full or partial profile)? | Methods, DCE design  (pg. 8-9) |
| 3.2 Was the number of profiles in each conjoint task justified? | Methods, DCE design  (pg. 9) |
| 3.3 Was (should) an opt-out or a status-quo alternative (be) included? | Methods, DCE design  (pg. 9) |
| 4. Was the choice of experimental design justified and evaluated? |  |
| 4.1 Was the choice of experimental design justified? Were alternative experimental designs considered? | Methods, DCE design  (pg. 8-9) |
| 4.2 Were the properties of the experimental design evaluated? | Methods, DCE design  (pg. 9) |
| 4.3 Was the number of conjoint tasks included in the data-collection instrument appropriate? | Methods, DCE design  (pg. 9) |
| 5. Were preferences elicited appropriately, given the research question? | Methods, Data collection (pg. 10-11) |
| 5.1 Was there sufficient motivation and explanation of conjoint tasks? | Methods, Data collection  (pg. 10-11) |
| 5.2 Was an appropriate elicitation format (that is, rating, ranking, or choice) used? Did (should) the elicitation format allow for indifference? | Methods, DCE design  (pg. 11) |
| 5.3 In addition to preference elicitation, did the conjoint tasks include other qualifying questions (for example, strength of preference, confidence in response, and other methods)? | Methods, Data collection (pg.11) |
| 6. Was the data collection instrument designed appropriately? |  |
| 6.1 Was appropriate respondent information collected (such as sociodemographic, attitudinal, health history or status, and treatment experience)? | Methods, Data collection (pg. 11) |
| 6.2 Were the attributes and levels defined, and was any contextual information provided? | Methods, Data collection (pg. 11) |
| 6.3 Was the level of burden of the data-collection instrument appropriate? Were respondents encouraged and motivated? | Methods, Data collection (pg. 11) |
| 7. Was the data-collection plan appropriate? | Methods, Data collection  (pg. 11) |
| 7.1 Was the sampling strategy justified (for example, sample size, stratification, and recruitment)? | Sample size and sampling strategy (pg. 9-10) |
| 7.2 Was the mode of administration justified and appropriate (for example, face-to-face, pen-and-paper, web-based)? | Methods, Data collection (pg. 10-11) |
| 7.3 Were ethical considerations addressed (for example, recruitment, information and/or consent, compensation)? | Ethics approval and consent (pg. 13) |
| 8. Were statistical analyses and model estimations appropriate? | Statistical Analysis  (pg. 11-13) |
| 8.1 Were respondent characteristics examined and tested? | Results, Participant characteristics (pg. 14) |
| 8.2 Was the quality of the responses examined (for example, rationality, validity, reliability)? | DCE Design (pg. 8-9) |
| 8.3 Was model estimation conducted appropriately? Were issues of clustering and subgroups handled appropriately? | Statistical Analysis  (pg. 12-13) |
| 9. Were the results and conclusions valid? | Results (pg. 14-17); Discussion (pg. 17-18) |
| 9.1 Did study results reflect testable hypotheses and account for statistical uncertainty? | Results (pg. 14-17) |
| 9.2 Were study conclusions supported by the evidence and compared with existing findings in the literature? | Discussion (pg. 18-19) |
| 9.3 Were study limitations and generalizability adequately discussed? | Discussion (pg. 20) |
| 10. Was the study presentation clear, concise, and complete? | Results (pg. 14-17) |
| 10.1 Was study importance and research context adequately motivated? | Introduction (pg. 5-7) |
| 10.2 Were the study data-collection instrument and methods described? | Methods, Data collection (pg. 10-11) |
| 10.3 Were the study implications clearly stated and understandable to a wide audience? | Discussion (pg. 17-20) |

## **Table S3. Preference weights from mixed effects models in seven countries**

| **Coefficients** | **Burkina Faso** | | **Nigeria** | | **Ethiopia** | | **Kenya** | | **Zambia** | | **Pakistan** | | **USA** |
| --- | --- | --- | --- | --- | --- | --- | --- | --- | --- | --- | --- | --- | --- |
| Opt-out | 0.18 (0.1, 0.25)*** | | 0.37 (0.31, 0.44)*** | | 0.19 (0.1, 0.28)*** | | 0.5 (0.43, 0.57)*** | | 0.04 (-0.03, 0.10) | | 1.96 (1.86, 2.07)*** | | 0.5 (0.42, 0.58)*** |
| **Format** |  | |  | |  | |  | |  | |  | |  |
| Pill | 0.43 (0.27, 0.59)*** | | 0.55 (0.40, 0.70)*** | | 0.2 (0.01, 0.40)* | | 0.07 (-0.07, 0.22) | | 0.26 (0.11, 0.4)*** | | -0.26 (-0.48, -0.05)* | | 0.78 (0.58, 0.98)*** |
| Injection | 0.01 (-0.19, 0.21) | | 0.26 (0.06, 0.46)* | | 0.64 (0.38, 0.90)*** | | 0.44 (0.25, 0.63)*** | | 0.39 (0.2, 0.58)*** | | 0.55 (0.29, 0.81)*** | | -0.09 (-0.33, 0.16) |
| Implant | 0.12 (0.01, 0.23)* | | 0.02 (-0.08, 0.13) | | 0.29 (0.15, 0.44)*** | | 0.2 (0.09, 0.31)*** | | 0.32 (0.21, 0.43)*** | | -0.18 (-0.38, 0) | | 0 (-0.14, 0.14) |
| Patch | 0.24 (0.16, 0.32)*** | | -0.17 (-0.24, -0.09)*** | | 0.4 (0.30, 0.50)*** | | 0.21 (0.13, 0.28)*** | | 0.29 (0.21, 0.36)*** | | 0.37 (0.27, 0.47)*** | | -0.14 (-0.25, -0.04)** |
| IUD | -0.34 (-0.44, -0.23)*** | | -0.32 (-0.42, -0.22)*** | | -0.75 (-0.90, -0.61)*** | | -0.40 (-0.50,-0.30)*** | | -0.58 (-0.69, -0.47)*** | | -0.62 (-0.81, -0.43)*** | | -0.16 (-0.29, -0.02)* |
| Ring | -0.46 (-0.57, -0.35)*** | | -0.34 (-0.45, -0.24)*** | | -0.78 (-0.95, -0.62)*** | | -0.52 (-0.64, -0.41)*** | | -0.67 (-0.79, -0.56)*** | | 0.15 (-0.01, 0.30) | | -0.39 (-0.54, -0.24)*** |
| **Dosing interval** |  | |  | |  | |  | |  | |  | |  |
| Short | -0.17 (-0.29, -0.05)** | | -0.05 (-0.17, 0.06) | | -0.09 (-0.25, 0.06) | | -0.09 (-0.21, 0.02) | | -0.13 (-0.24, -0.01)* | | 0 (-0.15, 0.15) | | -0.18 (-0.33, -0.04)* |
| Medium | 0.07 (-0.06, 0.21) | | 0.01 (-0.12, 0.14) | | 0.07 (-0.11, 0.24) | | 0.04 (-0.09, 0.17) | | 0.03 (-0.10, 0.16) | | -0.09 (-0.26, 0.08) | | 0.01 (-0.16, 0.17) |
| Long | 0.1 (0.02, 0.18)* | | 0.04 (-0.04, 0.12) | | 0.03 (-0.08, 0.13) | | 0.06 (-0.02, 0.14) | | 0.09 (0.01, 0.17)* | | 0.09 (-0.01, 0.19) | | 0.18 (0.08, 0.28)*** |
| **Who administers** |  | |  | |  | |  | |  | |  | |  |
| Self | -0.05 (-0.14, 0.04) | | -0.05 (-0.14, 0.03) | | 0.02 (-0.09, 0.13) | | -0.04 (-0.13, 0.05) | | 0.05 (-0.04, 0.14) | | -0.17 (-0.28, -0.06)** | | 0.16 (0.05, 0.27)** |
| HCW | -0.1 (-0.17, -0.03)** | | -0.03 (-0.10, 0.04) | | 0.01 (-0.08, 0.11) | | -0.03 (-0.1, 0.05) | | -0.11 (-0.18, -0.04)** | | 0.15 (0.05, 0.25)** | | -0.14 (-0.23, -0.05)** |
| HCW or self | 0.15 (0.02, 0.28)* | | 0.08 (-0.04, 0.21) | | -0.03 (-0.20, 0.13) | | 0.07 (-0.05, 0.19) | | 0.06 (-0.06, 0.18) | | 0.02 (-0.14, 0.18) | | -0.02 (-0.18, 0.14) |
| **Effect on menstruation** |  | |  | |  | |  | |  | |  | |  |
| Same as usual | -0.07 (-0.38, 0.25) | | 0.17 (-0.14, 0.47) | | 0.38 (-0.03, 0.80) | | 0.22 (-0.09, 0.52) | | 0 (-0.3, 0.31) | | 0.31 (-0.08, 0.70) | | 0.30 (-0.10, 0.70) |
| 3 months irregular,  then 1 week regular every month | 0.16 (-0.02, 0.35) | | 0 (-0.17, 0.18) | | -0.08 (-0.32, 0.16) | | 0.11 (-0.07, 0.29) | | 0.14 (-0.04, 0.31) | | -0.21 (-0.44, 0.02) | | 0.08 (-0.15, 0.31) |
| 3-6 months irregular,  then 1 week regular every month | 0.09 (-0.07, 0.25) | | -0.08 (-0.24, 0.07) | | -0.12 (-0.33, 0.08) | | -0.05 (-0.20, 0.10) | | -0.02 (-0.18, 0.13) | | -0.1 (-0.29, 0.1) | | -0.08 (-0.29, 0.13) |
| 3-6 months irregular, then none | -0.05 (-0.16, 0.05) | | -0.01 (-0.12, 0.09) | | 0 (-0.14, 0.13) | | -0.12 (-0.23, -0.02)* | | -0.05 (-0.16, 0.05) | | -0.07 (-0.20, 0.06) | | 0.10 (-0.03, 0.23) |
| Ongoing irregular | -0.14 (-0.25, -0.03)* | | -0.07 (-0.19, 0.04) | | -0.17 (-0.32, -0.02)* | | -0.15 (-0.26, -0.04)** | | -0.07 (-0.18, 0.04) | | 0.07 (-0.06, 0.19) | | -0.4 (-0.55, -0.26)*** |
| **Effect on weight** |  | |  | |  | |  | |  | |  | |  |
| No change | 0.07 (-0.01, 0.16) | | 0.11 (0.03, 0.19)** | | 0.13 (0.03, 0.24)* | | 0.11 (0.03, 0.19)** | | -0.11 (-0.19, -0.02)** | | 0.56 (0.44, 0.67)*** | | 0.42 (0.31, 0.53)*** |
| Gain up to 2.5 kg in one year | -0.03 (-0.08, 0.02) | | -0.02 (-0.07, 0.03) | | 0.05 (0, 0.11) | | -0.03 (-0.08, 0.02) | | 0.02 (-0.03, 0.07) | | -0.01 (-0.08, 0.05) | | -0.05 (-0.1, 0.01) |
| Gain up to 4.5 kg in one year | -0.04 (-0.15, 0.06) | | -0.09 (-0.19, 0.01) | | -0.19 (-0.32, -0.05)** | | -0.08 (-0.18, 0.02) | | 0.09 (-0.01, 0.19) | | -0.54 (-0.68, -0.41)*** | | -0.37 (-0.50,-0.24)*** |
| **Time from discontinuation**  **to return to fertility** | |  | |  | |  | |  | |  | |  | |
| Short | 0.03 (-0.05, 0.10) | | 0.13 (0.05, 0.20)*** | | 0.11 (0.02, 0.21)* | | -0.01 (-0.08, 0.06) | | -0.01 (-0.09, 0.06) | | 0.11 (0, 0.22)* | | 0.04 (-0.06, 0.15) |
| Medium | 0.03 (-0.03, 0.08) | | -0.02 (-0.07, 0.03) | | 0.02 (-0.04, 0.09) | | 0.01 (-0.04, 0.06) | | -0.04 (-0.09, 0.01) | | -0.04 (-0.10, 0.02) | | 0 (-0.06, 0.06) |
| Long | -0.05 (-0.14, 0.04) | | -0.11 (-0.20, -0.02)* | | -0.14 (-0.25, -0.02)* | | 0 (-0.08, 0.09) | | 0.05 (-0.03, 0.14) | | -0.07 (-0.19, 0.05) | | -0.04 (-0.15, 0.07) |
| **Interaction terms between format and dosing interval** |  | |  | |  | |  | |  | |  | |  |
| Pill: 24 hrs | -0.19 (-0.36, -0.01)* | | -0.04 (-0.21, 0.12) | | 0 (-0.22, 0.22) | | -0.01 (-0.17, 0.16) | | -0.19 (-0.36, -0.02)* | | -0.24 (-0.43, -0.04)* | | -0.01 (-0.24, 0.21) |
| Pill: 1 month | -0.12 (-0.36, 0.12) | | -0.09 (-0.32, 0.14) | | 0.2 (-0.09, 0.49) | | 0.07 (-0.16, 0.29) | | -0.09 (-0.32, 0.14) | | 0.11 (-0.18, 0.40) | | -0.11 (-0.41, 0.18) |
| Pill: 3 months | 0.31 (-0.02, 0.64) | | 0.13 (-0.19, 0.46) | | -0.2 (-0.64, 0.24) | | -0.06 (-0.37, 0.26) | | 0.28 (-0.04, 0.6) | | 0.13 (-0.28, 0.52) | | 0.13 (-0.30, 0.55) |
| Injection: 3 months | 0.07 (-0.05, 0.19) | | -0.01 (-0.12, 0.11) | | -0.11 (-0.25, 0.04) | | -0.15 (-0.26, -0.03)** | | 0.05 (-0.07, 0.17) | | -0.2 (-0.34, -0.05)** | | -0.05 (-0.19, 0.10) |
| Injection: 4 months | -0.22 (-0.46, 0.01) | | -0.11 (-0.33, 0.11) | | 0.14 (-0.15, 0.43) | | -0.05 (-0.26, 0.15) | | -0.12 (-0.33, 0.1) | | 0.03 (-0.23, 0.28) | | 0.01 (-0.27, 0.28) |
| Injection: 6 months | 0.15 (-0.03, 0.33) | | 0.12 (-0.05, 0.29) | | -0.03 (-0.25, 0.19) | | 0.2 (0.04, 0.36)* | | 0.07 (-0.1, 0.23) | | 0.17 (-0.03, 0.37) | | 0.04 (-0.16, 0.24) |
| Implant: 6 months | -0.31 (-0.57, -0.06)* | | -0.26 (-0.51, 0.00)* | | 0 (-0.32, 0.33) | | -0.02 (-0.26, 0.22) | | -0.18 (-0.43, 0.08) | | -0.06 (-0.35, 0.24) | | -0.26 (-0.56, 0.05) |
| Implant: 1 year | 0.42 (0.14, 0.69)** | | 0.24 (-0.04, 0.52) | | -0.06 (-0.42, 0.30) | | -0.01 (-0.27, 0.26) | | 0.15 (-0.13, 0.43) | | -0.24 (-0.57, 0.09) | | 0.26 (-0.06, 0.59) |
| Implant: 2 years | -0.1 (-0.27, 0.07) | | 0.01 (-0.16, 0.18) | | 0.05 (-0.18, 0.28) | | 0.02 (-0.15, 0.2) | | 0.02 (-0.15, 0.2) | | 0.29 (0.06, 0.53)* | | -0.01 (-0.23, 0.22) |
| Patch: 3 months | 0.01 (-0.3, 0.32) | | 0.05 (-0.26, 0.36) | | -0.15 (-0.55, 0.26) | | -0.12 (-0.41, 0.18) | | 0.06 (-0.23, 0.36) | | 0.22 (-0.13, 0.58) | | -0.44 (-0.82, -0.05)* |
| Patch: 4 months | 0.03 (-0.09, 0.15) | | -0.03 (-0.14, 0.09) | | 0.01 (-0.13, 0.16) | | 0.06 (-0.04, 0.18) | | -0.03 (-0.14, 0.09) | | -0.19 (-0.33, -0.05)** | | 0.15 (0.01, 0.29)* |
| Patch: 6 months | -0.04 (-0.29, 0.21) | | -0.02 (-0.28, 0.23) | | 0.13 (-0.2, 0.45) | | 0.05 (-0.19, 0.29) | | -0.03 (-0.28, 0.21) | | -0.04 (-0.33, 0.26) | | 0.29 (-0.03, 0.61) |
| IUD: 6 months | -0.59 (-1.01, -0.18)** | | -0.01 (-0.4, 0.38) | | -0.09 (-0.62, 0.44) | | -0.18 (-0.56, 0.21) | | -0.49 (-0.86, -0.11)* | | 0.5 (-0.02, 1.03) | | -0.37 (-0.87, 0.12) |
| IUD: 2 years | 0.41 (0.01, 0.81)* | | -0.04 (-0.42, 0.35) | | -0.16 (-0.68, 0.37) | | 0 (-0.38, 0.39) | | 0.32 (-0.06, 0.7) | | -0.48 (-0.99, 0.03) | | -0.17 (-0.66, 0.33) |
| IUD: 5 years | 0.19 (0, 0.38) | | 0.04 (-0.15, 0.23) | | 0.24 (-0.02, 0.50) | | 0.18 (0, 0.36) | | 0.17 (-0.02, 0.35) | | -0.03 (-0.24, 0.18) | | 0.54 (0.31, 0.77)*** |
| Ring: 1 month | -0.02 (-0.18, 0.15) | | -0.05 (-0.21, 0.11) | | -0.23 (-0.44, -0.02)* | | -0.09 (-0.26, 0.07) | | -0.02 (-0.17, 0.14) | | -0.24 (-0.43, -0.05)* | | 0.03 (-0.18, 0.23) |
| Ring: 3 months | -0.08 (-0.29, 0.13) | | 0.08 (-0.12, 0.28) | | 0.27 (-0.02, 0.55) | | 0.14 (-0.07, 0.35) | | -0.03 (-0.24, 0.17) | | 0.23 (-0.02, 0.48) | | -0.11 (-0.37, 0.16) |
| Ring: 1 year | 0.1 (-0.03, 0.23) | | -0.03 (-0.16, 0.10) | | -0.04 (-0.23, 0.15) | | -0.05 (-0.19, 0.09) | | 0.05 (-0.09, 0.19) | | 0.02 (-0.14, 0.17) | | 0.08 (-0.09, 0.25) |
| **Random effects coefficients (SD)** | |  | |  | |  | |  | |  | |  | |
| Injection | -0.67 (-0.76, -0.59)*** | | -0.63 (-0.71, -0.55)*** | | -0.6 (-0.71, -0.50)*** | | -0.67 (-0.75, -0.58)*** | | -0.72 (-0.8, -0.64)*** | | 0.83 (0.67, 0.98)*** | | 1.13 (1.03, 1.23)*** |
| Implant | -0.82 (-0.91, -0.72)*** | | -0.75 (-0.83, -0.67)*** | | 0.72 (0.62, 0.81)*** | | 0.73 (0.66, 0.81)*** | | 0.67 (0.59, 0.75)*** | | -0.91 (-1.11, -0.70)*** | | -1.27 (-1.38, -1.16)*** |
| Patch | 0.85 (0.79, 0.91)*** | | -0.82 (-0.89, -0.75)*** | | 0.85 (0.77, 0.93)*** | | 0.80 (0.73, 0.86)*** | | 0.85 (0.79, 0.92)*** | | 1.22 (1.1, 1.34)*** | | -1.41 (-1.50, -1.31)*** |
| IUD | -1.03 (-1.15, -0.91)*** | | -0.65 (-0.74, -0.56)*** | | -1.16 (-1.32, -1.00)*** | | -0.96 (-1.08, -0.85)*** | | -0.97 (-1.09, -0.86)*** | | -1.34 (-1.6, -1.09)*** | | -1.59 (-1.72, -1.44)*** |
| Ring | 0.67 (0.58, 0.76)*** | | 0.69 (0.63, 0.76)*** | | 0.99 (0.89, 1.09)*** | | 0.72 (0.63, 0.80)*** | | 0.77 (0.69, 0.85)*** | | 0.55 (0.41, 0.69)*** | | 1.56 (1.45, 1.67)*** |

* <0.05; ** <0.01; *** < 0.001

**Figure S9. Preference weights by contraceptive format and format-specific dosing interval.** Bars represent estimated preference weights, and the error bars indicate 95% confidence intervals. Positive values reflect higher relative preference, while negative values reflect lower relative preference compared to other method formats within each country.

## **Table S4.** **Preference weights from mixed effects models across seven countries, with random effects for format, effect on menstruation, and effect on weight.**

| **Coefficients** | **Burkina Faso** | | **Nigeria** | | **Ethiopia** | | **Kenya** | | **Zambia** | | **Pakistan** | | **USA** |
| --- | --- | --- | --- | --- | --- | --- | --- | --- | --- | --- | --- | --- | --- |
| Opt-out | 0.23 (0.16, 0.31)*** | | 0.39 (0.32, 0.46)*** | | 0.19 (0.1, 0.28)*** | | 0.52 (0.46, 0.59)*** | | 0.05 (-0.01, 0.12) | | 2.05 (1.95, 2.15)*** | | 0.53 (0.45, 0.62)*** |
| **Format** |  | |  | |  | |  | |  | |  | |  |
| Pill | 0.47 (0.31, 0.64)*** | | 0.56 (0.41, 0.71)*** | | 0.29 (0.09, 0.49)** | | 0.11 (-0.04, 0.25) | | 0.26 (0.11, 0.41)*** | | 0.09 (-0.11, 0.3) | | 0.72 (0.5, 0.94)*** |
| Injection | 0.09 (-0.12, 0.3) | | 0.25 (0.04, 0.45)* | | 0.59 (0.32, 0.86)*** | | 0.45 (0.26, 0.65)*** | | 0.45 (0.25, 0.65)*** | | 0.35 (0.09, 0.61)** | | -0.08 (-0.35, 0.19) |
| Implant | 0.06 (-0.05, 0.17) | | 0.05 (-0.06, 0.16) | | 0.33 (0.18, 0.49)*** | | 0.2 (0.09, 0.32)*** | | 0.29 (0.18, 0.4)*** | | -0.16 (-0.36, 0.05) | | 0.06 (-0.08, 0.2) |
| Patch | 0.16 (0.07, 0.24)*** | | -0.17 (-0.25, -0.09)*** | | 0.44 (0.32, 0.55)*** | | 0.18 (0.1, 0.26)*** | | 0.28 (0.19, 0.36)*** | | 0.26 (0.15, 0.37)*** | | -0.14 (-0.27, -0.01)* |
| IUD | -0.36 (-0.47, -0.26)*** | | -0.31 (-0.42, -0.2)*** | | -0.78 (-0.93, -0.63)*** | | -0.42 (-0.53, -0.31)*** | | -0.63 (-0.74, -0.53)*** | | -0.32 (-0.47, -0.18)*** | | -0.17 (-0.31, -0.02)* |
| Ring | -0.42 (-0.53, -0.31)*** | | -0.39 (-0.5, -0.27)*** | | -0.87 (-1.05, -0.69)*** | | -0.52 (-0.64, -0.4)*** | | -0.65 (-0.77, -0.53)*** | | -0.23 (-0.41, -0.04)* | | -0.4 (-0.55, -0.25)*** |
| **Dosing interval** |  | |  | |  | |  | |  | |  | |  |
| Short | -0.16 (-0.28, -0.04)** | | -0.05 (-0.16, 0.07) | | -0.11 (-0.27, 0.05) | | -0.09 (-0.21, 0.02) | | -0.13 (-0.25, -0.01)* | | -0.02 (-0.17, 0.13) | | -0.18 (-0.33, -0.02)* |
| Medium | 0.03 (-0.11, 0.17) | | 0.01 (-0.12, 0.15) | | 0.09 (-0.09, 0.27) | | 0.03 (-0.1, 0.16) | | 0.03 (-0.11, 0.17) | | -0.09 (-0.27, 0.08) | | 0.01 (-0.17, 0.18) |
| Long | 0.13 (0.05, 0.21)** | | 0.04 (-0.05, 0.12) | | 0.02 (-0.09, 0.14) | | 0.06 (-0.02, 0.14) | | 0.1 (0.01, 0.18)* | | 0.12 (0.01, 0.23)* | | 0.17 (0.06, 0.28)** |
| **Who administers** |  | |  | |  | |  | |  | |  | |  |
| Self | -0.02 (-0.11, 0.08) | | -0.05 (-0.14, 0.04) | | 0.04 (-0.08, 0.15) | | -0.04 (-0.13, 0.05) | | 0.06 (-0.03, 0.15) | | -0.1 (-0.21, 0.01) | | 0.21 (0.09, 0.32)*** |
| HCW | -0.06 (-0.14, 0.01) | | -0.05 (-0.12, 0.02) | | -0.01 (-0.11, 0.1) | | -0.02 (-0.1, 0.05) | | -0.09 (-0.16, -0.01)* | | 0.05 (-0.07, 0.16) | | -0.13 (-0.23, -0.04)** |
| HCW or self | 0.08 (-0.05, 0.21) | | 0.1 (-0.03, 0.23) | | -0.03 (-0.2, 0.14) | | 0.07 (-0.06, 0.19) | | 0.02 (-0.1, 0.15) | | 0.05 (-0.12, 0.22) | | -0.07 (-0.24, 0.09) |
| **Effect on menstruation** |  | |  | |  | |  | |  | |  | |  |
| Same as usual | 0.1 (-0.23, 0.42) | | 0.16 (-0.16, 0.48) | | 0.29 (-0.16, 0.73) | | 0.21 (-0.1, 0.53) | | 0.08 (-0.25, 0.41) | | 0.06 (-0.36, 0.48) | | 0.23 (-0.19, 0.65) |
| 3 months irregular,  then 1 week regular every month | 0.05 (-0.14, 0.24) | | 0 (-0.18, 0.18) | | 0 (-0.25, 0.24) | | 0.11 (-0.07, 0.3) | | 0.09 (-0.1, 0.27) | | 0 (-0.23, 0.24) | | 0.05 (-0.19, 0.28) |
| 3-6 months irregular,  then 1 week regular every month | -0.05 (-0.21, 0.12) | | -0.1 (-0.26, 0.06) | | -0.11 (-0.32, 0.11) | | -0.08 (-0.23, 0.07) | | -0.1 (-0.26, 0.07) | | 0.06 (-0.16, 0.27) | | -0.13 (-0.36, 0.09) |
| 3-6 months irregular, then none | 0.04 (-0.07, 0.15) | | -0.03 (-0.14, 0.08) | | -0.05 (-0.19, 0.1) | | -0.11 (-0.22, -0.01)* | | -0.02 (-0.13, 0.08) | | -0.15 (-0.3, -0.01)* | | 0.16 (0.02, 0.3)* |
| Ongoing irregular | -0.15 (-0.26, -0.03)* | | -0.04 (-0.16, 0.08) | | -0.12 (-0.28, 0.03) | | -0.14 (-0.25, -0.03)* | | -0.05 (-0.16, 0.06) | | 0.04 (-0.11, 0.18) | | -0.31 (-0.45, -0.17)*** |
| **Effect on weight** |  | |  | |  | |  | |  | |  | |  |
| No change | 0.11 (0.02, 0.2)* | | 0.11 (0.03, 0.2)** | | 0.11 (-0.01, 0.22) | | 0.11 (0.02, 0.19)* | | -0.11 (-0.19, -0.02)* | | 0.36 (0.24, 0.47)*** | | 0.47 (0.34, 0.59)*** |
| Gain up to 2.5 kg in one year | -0.03 (-0.08, 0.02) | | -0.04 (-0.09, 0.01) | | 0.07 (0.01, 0.13)* | | -0.03 (-0.08, 0.03) | | 0.03 (-0.02, 0.08) | | 0.06 (-0.03, 0.16) | | -0.04 (-0.09, 0.02) |
| Gain up to 4.5 kg in one year | -0.08 (-0.18, 0.03) | | -0.07 (-0.18, 0.03) | | -0.17 (-0.31, -0.04)* | | -0.08 (-0.19, 0.02) | | 0.08 (-0.03, 0.18) | | -0.42 (-0.57, -0.26)*** | | -0.43 (-0.57, -0.28)*** |
| **Time from discontinuation**  **to return to fertility** | |  | |  | |  | |  | |  | |  | |
| Short | 0.12 (0.04, 0.2)** | | 0.12 (0.05, 0.2)** | | 0.06 (-0.04, 0.16) | | 0.01 (-0.07, 0.09) | | 0 (-0.08, 0.09) | | 0.05 (-0.05, 0.16) | | 0.11 (-0.02, 0.23) |
| Medium | 0.04 (-0.02, 0.09) | | 0 (-0.06, 0.05) | | 0.03 (-0.04, 0.09) | | 0.01 (-0.04, 0.06) | | -0.04 (-0.09, 0.02) | | -0.03 (-0.09, 0.04) | | 0.01 (-0.05, 0.07) |
| Long | -0.16 (-0.25, -0.06)** | | -0.12 (-0.21, -0.03)** | | -0.09 (-0.21, 0.03) | | -0.02 (-0.11, 0.07) | | 0.03 (-0.06, 0.13) | | -0.03 (-0.15, 0.09) | | -0.12 (-0.25, 0.01) |
| **Interaction terms between format and dosing interval** |  | |  | |  | |  | |  | |  | |  |
| Pill: 24 hours | -0.1 (-0.28, 0.08) | | -0.04 (-0.22, 0.13) | | -0.15 (-0.38, 0.09) | | -0.03 (-0.2, 0.14) | | -0.21 (-0.38, -0.03)* | | -0.19 (-0.41, 0.03) | | -0.03 (-0.26, 0.21) |
| Pill: 1 month | -0.04 (-0.28, 0.2) | | -0.11 (-0.34, 0.12) | | 0.15 (-0.16, 0.44) | | 0.11 (-0.1, 0.34) | | 0 (-0.24, 0.23) | | -0.14 (-0.44, 0.16) | | -0.11 (-0.42, 0.21) |
| Pill: 3 months | 0.14 (-0.19, 0.48) | | 0.16 (-0.17, 0.49) | | 0 (-0.46, 0.46) | | -0.08 (-0.41, 0.23) | | 0.21 (-0.12, 0.54) | | 0.34 (-0.1, 0.76) | | 0.13 (-0.31, 0.57) |
| Injection: 3 months | 0.08 (-0.04, 0.2) | | 0.02 (-0.1, 0.14) | | -0.12 (-0.27, 0.03) | | -0.15 (-0.27, -0.04)* | | 0.02 (-0.1, 0.14) | | -0.09 (-0.25, 0.07) | | -0.08 (-0.23, 0.08) |
| Injection: 4 months | -0.24 (-0.48, 0) | | -0.13 (-0.36, 0.1) | | 0.21 (-0.08, 0.5) | | -0.07 (-0.28, 0.14) | | -0.07 (-0.29, 0.15) | | -0.07 (-0.35, 0.21) | | 0.02 (-0.27, 0.3) |
| Injection: 6 months | 0.16 (-0.02, 0.34) | | 0.11 (-0.06, 0.28) | | -0.09 (-0.31, 0.13) | | 0.22 (0.06, 0.38)** | | 0.05 (-0.12, 0.21) | | 0.16 (-0.04, 0.36) | | 0.06 (-0.14, 0.26) |
| Implant: 6 months | -0.43 (-0.7, -0.17)** | | -0.24 (-0.5, 0.01) | | 0.07 (-0.26, 0.41) | | -0.04 (-0.28, 0.21) | | -0.18 (-0.44, 0.08) | | -0.03 (-0.35, 0.28) | | -0.4 (-0.71, -0.09)* |
| Implant: 1 year | 0.39 (0.1, 0.68)** | | 0.26 (-0.03, 0.54) | | -0.04 (-0.41, 0.34) | | -0.01 (-0.29, 0.27) | | 0.1 (-0.19, 0.39) | | -0.11 (-0.46, 0.24) | | 0.43 (0.09, 0.78)* |
| Implant: 2 years | 0.04 (-0.13, 0.22) | | -0.01 (-0.19, 0.16) | | -0.04 (-0.29, 0.22) | | 0.05 (-0.13, 0.24) | | 0.08 (-0.11, 0.27) | | 0.14 (-0.1, 0.38) | | -0.04 (-0.27, 0.21) |
| Patch: 3 months | -0.35 (-0.76, 0.07) | | 0.03 (-0.36, 0.42) | | -0.2 (-0.75, 0.35) | | -0.13 (-0.52, 0.27) | | -0.38 (-0.78, 0.02) | | 0.18 (-0.33, 0.69) | | -0.18 (-0.72, 0.35) |
| Patch: 4 months | 0.09 (-0.32, 0.51) | | -0.04 (-0.44, 0.35) | | -0.04 (-0.59, 0.51) | | -0.06 (-0.47, 0.34) | | 0.18 (-0.23, 0.59) | | -0.2 (-0.71, 0.32) | | -0.27 (-0.8, 0.28) |
| Patch: 6 months | 0.25 (0.06, 0.45)** | | 0.01 (-0.18, 0.2) | | 0.23 (-0.03, 0.5) | | 0.18 (0, 0.36) | | 0.2 (0.01, 0.39)* | | 0.02 (-0.21, 0.26) | | 0.45 (0.22, 0.69)*** |
| IUD: 6 months | -0.15 (-0.47, 0.17) | | 0.04 (-0.28, 0.36) | | -0.04 (-0.47, 0.39) | | -0.11 (-0.42, 0.2) | | 0.01 (-0.3, 0.33) | | 0.17 (-0.21, 0.55) | | -0.38 (-0.78, 0.01) |
| IUD: 2 years | -0.03 (-0.15, 0.1) | | -0.02 (-0.14, 0.1) | | -0.01 (-0.17, 0.15) | | 0.04 (-0.07, 0.16) | | -0.06 (-0.18, 0.06) | | -0.08 (-0.23, 0.06) | | 0.07 (-0.08, 0.22) |
| IUD: 5 years | 0.18 (-0.07, 0.43) | | -0.02 (-0.28, 0.24) | | 0.05 (-0.3, 0.4) | | 0.07 (-0.18, 0.33) | | 0.04 (-0.21, 0.31) | | -0.08 (-0.39, 0.24) | | 0.31 (-0.01, 0.65) |
| Ring: 1 month | -0.02 (-0.18, 0.15) | | -0.09 (-0.25, 0.08) | | -0.23 (-0.45, -0.02)* | | -0.1 (-0.27, 0.06) | | -0.04 (-0.19, 0.12) | | -0.17 (-0.37, 0.03) | | 0 (-0.21, 0.2) |
| Ring: 3 months | 0.02 (-0.2, 0.24) | | 0.12 (-0.09, 0.32) | | 0.26 (-0.02, 0.55) | | 0.17 (-0.04, 0.38) | | 0.03 (-0.19, 0.24) | | 0.04 (-0.23, 0.3) | | -0.11 (-0.38, 0.16) |
| Ring: 1 year | 0 (-0.14, 0.14) | | -0.03 (-0.16, 0.11) | | -0.03 (-0.22, 0.17) | | -0.06 (-0.21, 0.08) | | 0.01 (-0.13, 0.15) | | 0.13 (-0.04, 0.31) | | 0.11 (-0.06, 0.28) |
| **Random effects coefficients (SD)** | |  | |  | |  | |  | |  | |  | |
| **Format** |  | |  | |  | |  | |  | |  | |  |
| Injection | -0.68 (-0.76, -0.59)*** | | -0.61 (-0.71, -0.51)*** | | -0.61 (-0.72, -0.51)*** | | -0.66 (-0.75, -0.56)*** | | -0.74 (-0.82, -0.66)*** | | 0.43 (0.19, 0.68)*** | | 1.12 (1, 1.24)*** |
| Implant | -0.71 (-0.83, -0.58)*** | | -0.73 (-0.82, -0.65)*** | | 0.68 (0.58, 0.78)*** | | 0.69 (0.61, 0.78)*** | | 0.65 (0.56, 0.73)*** | | -0.76 (-1.02, -0.5)*** | | -1.19 (-1.3, -1.07)*** |
| Patch | 0.76 (0.7, 0.83)*** | | -0.84 (-0.91, -0.76)*** | | 0.83 (0.74, 0.91)*** | | 0.79 (0.72, 0.87)*** | | 0.84 (0.78, 0.91)*** | | 1.09 (0.94, 1.24)*** | | -1.29 (-1.42, -1.17)*** |
| IUD | -0.88 (-1.03, -0.73)*** | | -0.66 (-0.79, -0.52)*** | | -1.16 (-1.32, -1)*** | | -0.97 (-1.09, -0.84)*** | | -0.98 (-1.08, -0.87)*** | | -0.57 (-0.85, -0.28)*** | | -1.47 (-1.7, -1.25)*** |
| Ring | 0.64 (0.52, 0.76)*** | | 0.72 (0.65, 0.78)*** | | 1.02 (0.91, 1.13)*** | | 0.72 (0.6, 0.84)*** | | 0.78 (0.7, 0.87)*** | | -0.61 (-0.81, -0.4)*** | | 1.49 (1.35, 1.64)*** |
| **Effect on menstruation** |  | |  | |  | |  | |  | |  | |  |
| 3 months irregular,  then 1 week regular every month | -0.02 (-0.11, 0.08) | | -0.04 (-0.09, 0.01) | | 0.02 (-0.06, 0.11) | | 0.04 (-0.02, 0.11) | | 0.14 (0.02, 0.26)* | | 0.04 (-0.34, 0.43) | | 0.09 (-0.06, 0.24) |
| 3-6 months irregular,  then 1 week regular every month | -0.49 (-0.55, -0.43)*** | | -0.4 (-0.45, -0.35)*** | | -0.39 (-0.49, -0.3)*** | | -0.33 (-0.4, -0.26)*** | | -0.4 (-0.46, -0.33)*** | | -0.5 (-0.6, -0.4)*** | | 0.44 (0.33, 0.56)*** |
| 3-6 months irregular, then none | -0.27 (-0.41, -0.12)*** | | 0.22 (0.07, 0.36)** | | 0.47 (0.34, 0.59)*** | | -0.39 (-0.52, -0.26)*** | | 0.04 (-0.22, 0.29) | | -0.36 (-0.58, -0.13)** | | -0.47 (-0.65, -0.29)*** |
| Ongoing irregular | 0.12 (-0.01, 0.26) | | 0.01 (-0.26, 0.28) | | 0.19 (-0.03, 0.43) | | -0.02 (-0.13, 0.1) | | 0.27 (0.15, 0.39)*** | | -0.3 (-0.59, -0.01)* | | 0.16 (0.01, 0.32)* |
| **Effect on weight gain** |  | |  | |  | |  | |  | |  | |  |
| Gain up to 2.5 kg in one year | -0.61 (-0.68, -0.54)*** | | -0.15 (-0.43, 0.13) | | 0.13 (-0.08, 0.35) | | -0.08 (-0.56, 0.4) | | 0.1 (-0.26, 0.47) | | -0.94 (-1.1, -0.78)*** | | -0.64 (-0.86, -0.42)*** |
| Gain up to 4.5 kg in one year | 0.2 (0.11, 0.3)*** | | -0.3 (-0.39, -0.21)*** | | -0.14 (-0.27, -0.01)* | | -0.25 (-0.34, -0.16)*** | | -0.09 (-0.27, 0.08) | | -0.64 (-0.83, -0.45)*** | | -0.11 (-0.31, 0.09) |

* <0.05; ** <0.01; *** < 0.001

**Figure S10. Preference weights for six contraceptive attributes by country from mixed effects models with random effects for format, effect on menstruation, and effect on weight.** Bars represent estimated preference weights, and the error bars indicate 95% confidence intervals. Positive values reflect stronger preference, while negative values reflect weaker preference compared to the average preference across all levels in a given attribute.

1. Orme B. Sample size issues for conjoint analysis studies. Sequim: Sawtooth Software Technical Paper, 1998.
2. Johnson R, Orme B. Getting the most from CBC. Sequim: Sawtooth Software Research Paper Series, Sawtooth Software; 2003.
